# Phagotrophic protists preserve antibiotic-resistant opportunistic human pathogens in the vegetable phyllosphere

**DOI:** 10.1038/s43705-023-00302-z

**Published:** 2023-09-02

**Authors:** Chenshuo Lin, Li-Juan Li, Kexin Ren, Shu-Yi-Dan Zhou, Alain Isabwe, Le-Yang Yang, Roy Neilson, Xiao-Ru Yang, Eddie Cytryn, Yong-Guan Zhu

**Affiliations:** 1 Key Laboratory of Urban Environment and Health, Institute of Urban Environment, Chinese Academy of Sciences, 1799 Jimei Road, Xiamen, 361021 China; 2 University of Chinese Academy of Sciences, 19A Yuquan Road, 100049 Beijing, China; 3 Key Laboratory of Vegetation Restoration and Management of Degraded Ecosystems, South China Botanical Garden, Chinese Academy of Sciences, Xingke Road 723, Tianhe District, Guangzhou, 510650 China; 4 Ecological Sciences, The James Hutton Institute, Dundee, DD2 5DA Scotland UK; 5 Department of Soil Chemistry, Plant Nutrition and Microbiology, Institute of Soil, Water and Environmental Sciences, The Volcani Institute, Agriculture Research Organization, 7528809 Rishon Lezion, Israel; 6 State Key Laboratory of Urban and Regional Ecology, Research Center for Eco-Environmental Sciences, Chinese Academy of Sciences, 100085 Beijing, China

**Keywords:** Environmental sciences, Environmental microbiology, Genomics

## Abstract

Food safety of leafy greens is an emerging public health issue as they can harbor opportunistic human pathogens (OHPs) and expose OHPs to consumers. Protists are an integral part of phyllosphere microbial ecosystems. However, our understanding of protist-pathogen associations in the phyllosphere and their consequences on public health remains poor. Here, we examined phyllosphere protists, human pathogen marker genes (HPMGs), and protist endosymbionts from four species of leafy greens from major supermarkets in Xiamen, China. Our results showed that *Staphylococcus aureus* and *Klebsiella pneumoniae* were the dominant human pathogens in the vegetable phyllosphere. The distribution of HPMGs and protistan communities differed between vegetable species, of which Chinese chive possessed the most diverse protists and highest abundance of HPMGs. HPMGs abundance positively correlated with the diversity and relative abundance of phagotrophic protists. Whole genome sequencing further uncovered that most isolated phyllosphere protists harbored multiple OHPs which carried antibiotic resistance genes, virulence factors, and metal resistance genes and had the potential to HGT. *Colpoda* were identified as key phagotrophic protists which positively linked to OHPs and carried diverse resistance and virulence potential endosymbiont OHPs including *Pseudomonas nitroreducens*, *Achromobacter xylosoxidans*, and *Stenotrophomonas maltophilia*. We highlight that phyllosphere protists contribute to the transmission of resistant OHPs through internalization and thus pose risks to the food safety of leafy greens and human health. Our study provides insights into the protist-OHP interactions in the phyllosphere, which will help in food safety surveillance and human health.

## Introduction

Consuming raw or minimally processed leafy greens has become a popular healthy lifestyle choice for consumers [1]. However, the vegetable phyllosphere is a known reservoir of microbes [2] that can also host human pathogens without exhibiting any sign of spoilage or deterioration of the phyllosphere tissues, raising concerns about food safety [3]. Several outbreaks of severe food poisoning have been attributed to the consumption of raw leafy greens contaminated by pathogenic microorganisms, such as *Salmonella* spp., *E. coli*, and *Listeria monoctogenes* [4, 5]. According to the USDA Economic Research Service in 2016, consumers tend to believe that leafy greens produced with organic fertilizer (usually animal manure) without pesticides are safer and healthier than conventionally produced leafy greens [6]. However, they may ignore the potential health risks of organic vegetables, such as increased ARGs and human pathogens in leafy greens due to the application of animal manure [7]. Therefore, leafy greens especially organically produced ones are potential hot spots for the transmission of food-borne opportunistic human pathogens (OHPs) [8–10] in light of the “One Health” concept.

Protists are one of the overlooked phyllosphere microbes that potentially affect human health by their potential pathogenicity [11] or shaping phyllosphere bacterial composition and function [12] (including bacterial human pathogens). Some phyllosphere protists are responsible for causing human diseases, including *Acanthamoeba* spp., *Giardia duodenalis*, *Entamoeba* spp., *Cryptosporidium*, etc. [13, 14]. More than 100 diverse environmental amoebae were reported to reduce the number of OHPs such as *Acinetobacter baumannii*, *Klebsiella pneumoniae, Pseudomonas aeruginosa*, and *Staphylococcus aureus* [15, 16] by phagocytosis, killing OHPs intracellularly with toxic materials such as copper and arsenic [17]. In response to predation stress, specific pathogens evolve virulence (e.g., Shiga toxin and Violacein) [18–21] or mental resistance (e.g., copper resistance) [22] to resist digestion or internal shuttling to the contractile vacuole to replicate before being exocytosed to the environment [23]. Efflux pump systems closely associated with antibiotic resistance are also involved in grazing defense strategies. For example, the *C. jejuni* RND-type efflux pump, CmeABC, associated with multidrug resistance, may be involved in virulence and survival in *A. polyphaga* [24]. Meanwhile, protist-adapted pathogens may possess enhance environmental persistence, resistance, and transmission, thus enhancing the colonization in eukaryotic organisms of these pathogens [25]. For example, *Vibrio cholerae* that grew intracellularly in *Acanthamoeba castellaniiprotists* displayed *flrA* mutations that increased the competitive fitness of *V.cholerae* and colonization potential in zebrafish [26]. Similarly, amoeba-adapted *L. pneumophila* showed increased resistance to antibiotics and chlorine and were more infectious than in vitro-grown *L. pneumophila* [27]. Therefore, protists can be “Trojan horses” (reservoir, shelters, and vectors) and “training grounds” that select for enhanced survival and virulence traits of OHPs [28–31]. To date, researches on phyllosphere protists in leafy greens have mainly focused on pathogens [32, 33]. Studies evaluating the endosymbiotic mechanisms of protists are generally based on co-cultivation assays between a single protist host and a specific pathogen [34, 35]. Therefore, our understanding of *in situ* pathogen-protist associations in the vegetable phyllosphere and their possible impact on human health remains poor. It is also not clear to us whether the composition of the protist community and its relationship with OHPs are different in the phyllosphere of organically and conventionally produced vegetables.

To reveal the associations between phyllosphere protists and OHPs in leafy greens, we characterized the protistan community in four vegetable species that applied two different production systems (organic vs conventional) sourced from four major supermarket chains in Xiamen, China. Subsequently, we quantified selected human pathogen marker genes (HPMGs) in the phyllosphere of leafy greens using high-throughput quantitative PCR (HT-qPCR) and investigated the correlations between protists and HPMGs. Finally, we isolated endosymbionts of phyllosphere protists and identified their potential pathogenicity, virulence, and resistance traits using whole genome sequencing.

## Materials and methods

### Sample collection

To investigate the microbial communities of vegetable phyllosphere, we collected four species of both conventionally produced leafy greens (CPLGs) and organically produced leafy greens (OPLGs), including Chinese chive (*Allium tuberosum*), Chinese cabbage (*Brassica rapa subsp. pekinensis*), cabbage (*Brassica Oleracea var. capitata)*, and lettuce *(Lactuca sativa)* with three replicates of each during September and November 2020 from four supermarket chains located in Xiamen city, China (24°48′N, 118°08′E). All organically produced leafy greens are China Organic Standard GB/T 19630-2019 certified products. The China Organic Standard GB/T 19630-2019 certification guarantees the absence of growth regulating agents, chemical and synthetic products including pesticides, etc., and the presence of a strict organic management system. This standard recommends the use of biofertilizers from organic sources, which are mainly derived from animal manures or manures mixed with crop residues in China. Chemical fertilizer has been reported to affect some specific microbial taxa in the phyllosphere by altering photosynthetic activity and carbon assimilation by leaves, leading to changing available resources on the leaf, which are thought to shape the phyllosphere microbiome [36]. Before being transported by cleaned specialized transportation, organically produced vegetables should be cleaned, sorted, preserved, etc. by cleaned-up equipment to avoid contamination. The Chinese chives and cabbage were grown in Putian, Fujian, whereas the lettuce and Chinese cabbage were from Zhangjiakou, Hebei. The OPLGs were wrapped, packaged in sealed plastic bags and refrigerated (~4 °C), the CPLGs were not processed or packaged and displayed at room temperature. A total of 96 samples were immediately transferred to the lab and processed as follows.

After removing the outermost leaves, approximately 60 g of fresh vegetable leaves (both spear leaves and mature leaves) from each sample were placed into individual conical flasks (250 mL) containing 150 mL 0.01 M sterile buffer solution (120 mg NaCl, 4 mg MgSO_4_·7H_2_O, 4 mg CaCl_2_·2H_2_O, 359 mg Na_2_HPO_4_·12H_2_O, and 130 mg KH_2_PO_4_ in 1 L of deionized water) and shaken (180 rpm) at 30 °C for 2 h. The suspensions were then sonicated for 10 min and then divided equally into two groups, one for DNA extraction and the other for protists enrichment. Thereafter, each sample was filtered through a 0.22-µm cellulose membrane to collect the microbial community.

### DNA extraction, PCR assays, and amplicon sequencing

Membranes with adhering microbes were cut into small pieces with a sterilized cutter. DNA was extracted from them using the FastDNA^®^ Spin Kit for Soil (MP Biomedical, Santa Ana, CA), following the instructions listed by the manufacturer. To assess the concentration and quality of DNA, a NanoDrop Spectrophotometer (Nanodrop ND-1000, Thermo Scientific, Waltham, MA) and 1.0% agarose gel electrophoresis were used. DNA was stored at −20 °C until further use.

The V4 region of the eukaryotic 18S rRNA gene was amplified using the primer set TAReukFWD1F and TAReukREV3R [37]. To avoid amplification of chloroplast sequences [38], the V5-V7 region of bacterial 16S rRNA gene was selected for amplification with primers pair 799F and 1193R [39]. The protocols describing the eukaryotic 18S rRNA gene and bacterial 16S rRNA gene amplifications are detailed in Supplementary Methods S1. High-throughput sequencing was performed on an Illumina MiSeqPE300 sequencer (Majorbio, Shanghai, China).

### Sequencing processing

After sequencing, Illumina data was processed using the QIIME2 pipeline (version 2018.11, https://qiime2.org) [40]. Amplicon sequence variants (ASVs) were identified using DADA2 [41]. All eukaryotic ASVs were taxonomically assigned using the Protist Ribosomal Reference (PR2) database version 4.14.0 (https://github.com/pr2database/pr2database) [42] and blast-2.9.0 (http://ftp.ncbi.nlm.nih.gov/blast/executables/blast+/2.9.0/). Bacterial taxonomic identity was determined using the Silva 138.1 database (https://www.arb-silva.de/) [43]. After removing mitochondria, chloroplast, Fungi, Metazoa, Rhodophyta, Streptophyta, unclassified Opisthokonta, and embryophyceae sequences [44–46], and samples with less than 455 protist sequences (the minimum number of sequences in samples to reach the plateau based on the rarefaction curve Supplementary Fig. S1), we retained 209,942 valid protist sequences assigned to 640 ASVs in 72 samples. ASVs with an identity value lower than 80% were discarded. Here, we defined core bacteria as bacterial ASVs that presenting in all samples.

### Cultivation of phyllosphere protists

Phyllosphere-derived protists collected on the membranes described above were repeatedly washed with 5 ml 0.01 M sterile Page’s amoeba saline (PAS) (Supplementary Fig. S2A). Protists were isolated by serial dilution with PAS in the resulting suspensions of three replicates and were fed with (OD_600_ 1.5) heat-inactivated *E. coli* (DH5α) in 96-well plates at 28 °C in the dark, which inevitably selects against non-bacterivorous and small protistan taxa [47]. Microscopic observations were performed daily for the presence of phyllosphere protists and their morphology and locomotion by an inverted microscope with 400 times magnification (Nikon Eclipse Ti-U, Tokyo, Japan). Each isolated protist was then subcultured three times in fresh PAS medium with heat-inactivated *E. coli* every 4–7 days. Subculture might lower the presence of false endosymbionts (e.g. incompletely digested bacteria or bacteria that accidentally appear within protists) despite the inevitable possibility of preferential enrichment. Protists were then identified via 18S rRNA gene sequencing using the PSSU (5′-CTT TCG ATG GTA GTG TAT TGG ACT AC-3′) and EukB (5′-TGATCC TTC TGC AGG TTC ACC TAC-3′) primers [48, 49] (Supplementary Table S1). A maximum likelihood tree was constructed using the 18S rRNA gene sequences of phyllosphere protists isolated in this study, together with eight reference sequences downloaded from the NCBI GenBank using IQ-TREE (http://iqtree.cibiv.univie.ac.at/) with 1000 bootstrap iterations.

### Isolation of bacterial endosymbionts in phyllosphere protists

To obtain the bacterial endosymbionts that can stably survive in the protists, protists were separated from extracellular bacteria using S3e Cell Sorter (Bio-Rad, USA) equipped by 488 nm laser systems as previously described [44]. Briefly, protists were filtered through a sterile nylon 100 μm cell strainer (Solarbio, China) to avoid plugging the cell sorter before sorting. A green fluorescing probe Lysotracker Green DND-26 fluorescent signal was excited by a 488 nm laser and collected in bandpass Filter1 (525/30 nm). Protists stained with Lysotracker Green DND-26 (75 nmol/L; Invitrogen, USA) [50] were sorted with a purity yield mode based on the higher internal complexity (side scatter, SSC) versus bigger cell size (forward scatter, FSC) as well as higher green fluorescence compared to bacteria [50] (Supplementary Fig. S2B). Sorted protists were confirmed by an inverted microscope (Nikon Eclipse Ti-U, Tokyo, Japan) immediately. To further kill extracellular bacteria without harming the protists and their bacterial endosymbionts, sorted protists were then incubated for 1 h with 50 µg ml^−1^ each of gentamicin, ampicillin, and meropenem [51] at room temperature. After rinsing twice with PAS, antibiotic-treated protists were passed through flow cytometry again to further remove extracellular bacteria. Each sample had four replicates. Sorted protists were collected by centrifuging at 400 ×g for 2 min and were lysed with 0.4% Triton X-100 to release the intracellular bacteria cells from protists in three replicates. Then, 100 μL of lysates were plated on LB agar plates [22, 51] in triplicate and incubated for 36 h at 37 °C. Samples not amended with 0.4% Triton X-100 served as a control to validate that there were no extracellular bacteria.

### Genome sequencing and analyses

Finally, a total of 206 single-colony bacterial endosymbionts were obtained and identified by PCR using 16S rRNA gene (27F and 1492R) primers. By blasting the full-length 16S rRNA gene against the NCBI-nr database, 50 different endosymbionts were selected. Genomic DNA of 50 bacterial endosymbionts was extracted using the Wizard^®^ Genomic DNA Purification Kit (Promega) according to the manufacturer’s instructions and quantified by TBS-380 fluorometer (Turner BioSystems Inc., Sunnyvale, CA). Sequencing libraries were then sequenced at Shanghai Majorbio Bio-pharm Technology Co., Ltd, using the Illumina HiSeq X platform that generated paired-end reads of 150 bp. Genome sequences were de novo assembled using SPAdes v.3.6.2. We successfully obtained a total of 36 high-quality genomes of bacterial endosymbionts. Open reading frames (ORFs) were predicted using Prodigal v.2.6.3 [52]. Taxonomy affiliation was determined by MetaPhlAn2 [53]. Marker genes of isolated bacterial endosymbionts and 38 reference genomes (30 clinical genomes and eight environmental genomes) downloaded from the NCBI GenBank were used to build a maximum likelihood tree by IQ-TREE (http://iqtree.cibiv.univie.ac.at/) with 1000 bootstrap iterations. The OHPs were defined according to the A-to-Z database and previous reports (Table S2). Antibiotic resistance genes (ARGs) and metal resistance genes (MRGs) were identified by comparing the amino acid sequence of each gene with sequences in the CARD database [54] and the BacMet predicted database (version 1.1; http://bacmet.biomedicine.gu.se) [55] with an e-value ≤ 10^−10^, identity ≥80% and a minimal coverage ≥70% [56], respectively. Virulence factor genes (VFGs) were identified with BLASTP (e-value ≤ 10^−10^ with minimal identity and coverage of 80%) homology searches against the VFDB dataset, which includes only genes associated with experimentally verified virulence factors [57]. PlasFlow was used to predict plasmid sequences from ARG-carrying contigs [58]. ICEfinder online tool was used to estimate the transferability of ARGs involving integrative and conjugative elements (ICEs) and integrative and mobilizable elements (IMEs) [59]. ARGs have the potential to be transmitted if mobile genetic components (MGEs) are present within 10 open reading frames upstream or downstream of ARGs in the same contig [60].

### HT-qPCR assays

A previously described TaqMan probe-based HT-qPCR method using the WaferGen SmartChip Real-Time PCR system platform (WaferGen Inc. USA) [61], was used to quantify human pathogen genes. A total of 68 primer pairs targeting 33 human pathogens were used in addition to a universal primer pair and probe of bacterial 16S rRNA gene to act as a reference gene (Supplementary Table S3). HT-qPCR assays were performed in triplicate using the TaqMan^®^ Gene Expression Master Mix kit. More information about amplification conditions is available in Supplementary Methods S2. The absolute abundance (copies/uL) of microbial markers was calculated according to standard curves (Supplementary Table S4).

### Statistical analysis

The α-diversity of bacterial and protistan communities was estimated using: the Shannon index and the number of ASVs using QIIME2. Protistan trophic groups were assigned including phagotrophic protist, phototrophic protist, plant parasitic protist, animal parasitic protist, and human parasitic protist based on functional guilds at the genus level [62, 63]. One-way analysis of variance (ANOVA) was used to test differences in α-diversity using SPSS. Venn diagrams were created using the suite of “OmicStuido” online tools (https://www.omicstudio.cn/tool/) [64]. Permutational multivariate analysis of variance (PERMANOVA) was performed using the Adonis function with 999 permutations and sample ordination was visualized in a Principal coordinate analysis (PCoA). Heatmaps were performed by the “vegan” package in R 3.4.4 [65]. We evaluated the relationship between the absolute abundance of Top4 pathogen marker genes and microbial diversity (independently for bacteria, phagotrophic protists, and phototrophic protists) by linear regression. Random forest analysis was then conducted to identify predictors of the absolute abundance of prevalent pathogen marker genes using the “randomForest” R package [66]. To test the relationships between the diversity (observed richness and Shannon index) of microbe (bacteria and phagotrophic/phototrophic protists) or relative abundance of phyllosphere phagotrophic/phototrophic protists and the overall abundance or diversity of HPM, linear regression model, Pearson correlations, and associated significance were conducted with lm() function in R [67].

Co-occurrence networks were used to identify potential interactions between protists and bacteria for each studied leafy green. Abundant protistan and bacterial ASVs (i.e., with average relative abundance >0.1% and occurring in >30% of samples) were selected for the construction of co-occurrence networks. A pairwise Spearman correlation matrix was calculated using the “psych” package of R (Spearman; P. adjust method: FDR). Spearman correlation coefficients of (ρ) >0.5 (or < −0.5) with *p* < 0.05 were selected for the microbial network analysis to include a range of interaction strengths (not only strong interactions) [68]. Network properties were characterized and visualized using Gephi 0.9.2. The relationship between microbial nodes and quantified HPMGs was assessed using robust Spearman correlation coefficients of (ρ) >0.7 (or < −0.7) and *p* < 0.01 [69, 70]. We focused on the association between microbial nodes and HPMGs and omitted the connections within the microbes or HPMGs. The roles of nodes in the bacteria-HPMG-protist network were assigned based on the method proposed by Guimera and Amaral (2005) [71]. Roles included: within-module degree (*Zi*) which measures how well a particular feature is connected to others in the same module, and among-module connectivity or participation coefficient (*Pi*), which measures how a feature is linked to other modules in the network. Nodes were classified as peripherals, hubs, and connectors iterating their roles in the whole microbial network as previously described [72].

## Results

### Phyllosphere protistan and bacterial communities across vegetable species

**Fig. 1:**
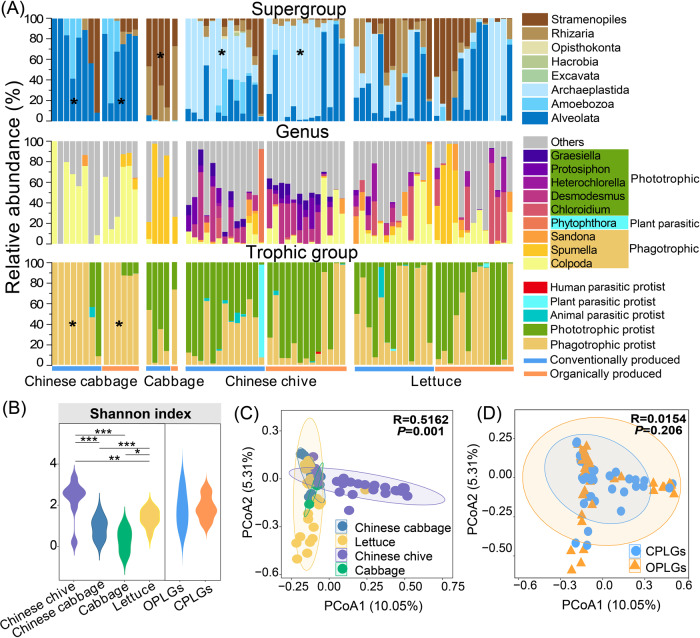
Distribution and taxonomic composition of phyllosphere protists in the leafy greens. **A** Protistan community composition of vegetable species presenting the proportion of the different protist supergroups, genera, and trophic groups. **B** Diversity index of protistan community across different vegetable species and different production systems (OPLGs vs CPLGs). **C** Principal coordinate analysis showing the distribution pattern of phyllosphere protistan communities in the four studied vegetable species and **D** different production systems. Significant differences in protistan taxa and diversity (compared to other vegetables) across vegetable species are indicated with asterisks (sign test, *p < 0.05, **p < 0.01, ***p < 0.001). “OPLGs” re*p*resents organically produced leafy greens and “CPLGs” represents conventionally produced leafy greens.

For all vegetable species, Alveolata (35.1%), Archaeplastida (32.6%), and Stramenopiles (17.2%) were the dominant protistan supergroup while *Colpoda* (phagotrophic; 16.1%), *Spumella* (phagotrophic), and *Desmodesmus* (phototrophic) were the Top 3 dominant genus (Fig. 1A). In terms of trophic groups, phototrophic protists (44.0%) characterized by Archaeplastida, and phagotrophic protists (44.8%) had comparable relative abundance (Fig. 1A). Colpodea (Ciliophora, Alveolata) was the main phagotrophic protistan class in Chinese chives, Chinese cabbages, and lettuces, while Filosa-Sarcomonadea (Cercozoa, Rhizaria) was the dominant phagotrophic protistan class in cabbages. Chrysophyceae (Ochrophyta; Stramenopiles) was the main phototrophic protistan classes in both Chinese cabbage and Cabbage samples, while Chlorophyceae (Chlorophyta; Archaeplastida) and Trebouxiophyceae (Chlorophyta; Archaeplastida) were the dominant phototrophic protistan classes in Chinese chives and lettuces, respectively (Supplementary Fig. S2). Animal parasite Apicomplexa was the most frequent parasite presenting in 29.2% of phyllosphere samples. Only one human parasite monocyte was found in one Chinese chive sample, with a relative abundance of 2.2% (Fig. 1A). There was no significant difference in protistan diversity or community composition between organic *vs*. conventional production systems (Fig. 1B, D). However, protistan richness (Fig. 1B; *p* < 0.05) and community composition (Fig. 1C, *p* = 0.001) differed significantly across the four analyzed vegetable species. Chinese chive had the highest protist diversity (Fig. 1B, *p* < 0.01), with the highest abundance of Archaeplastida (Ochrophyta, phototrophic protists; Fig. 1A, *p* < 0.05). Cabbage showed the lowest protistan richness (Fig. 1B) but higher relative abundances of Stramenopiles than other vegetables. Chinese cabbage had the highest relative abundances of phagotrophic protists and Alveolata (Fig. 1A, *p* < 0.05). We identified no shared protistan ASVs among vegetable species, but each vegetable had its unique protistan ASVs (Supplementary Fig. S4A).

In OPLGs and CPLGs, core bacterial ASVs represented 45.2% and 51.9% of the total phyllosphere bacteria, respectively (Supplementary Fig. S4B). Proteobacteria and Bacteroidetes were the most dominant bacterial phyla accounting for more than 75% of phyllosphere bacteria, with *Chryseobacterium* and *Pseudomonas* being the two most abundant genera (Supplementary Fig. S5C). As with protists, bacterial community composition differed between vegetable species (Supplementary Fig. S5A, *p* = 0.001). Chinese chive and lettuce had the highest bacterial richness (Supplementary Fig. S5B). The relative abundance of *Allorhizobium-Neorhizobium-Pararhizobium-Rhizobium* and *Curtobacterium* was significantly higher in Chinese chive and Chinese cabbage than in other vegetables, respectively (Supplementary Fig. S5C, *p* < 0.05). Production processes had a slight effect on the phyllosphere bacterial community composition and diversity (Supplementary Figs. S5A and S5B).

Not surprisingly, phyllosphere microbial networks also differed across vegetable species (Supplementary Fig. S6A). Chinese chive had more negative edges and the most complex network (highest number of nodes, edges, and average degree) followed by lettuces, while neither cabbage nor Chinese cabbage networks had protistan nodes (Supplementary Fig. S6B).

### Correlations between human pathogens and phyllosphere protists

**Fig. 2:**
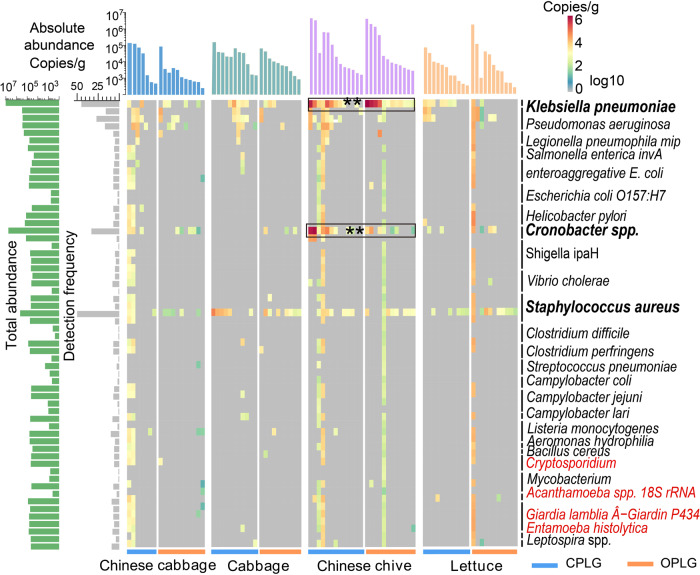
Absolute abundance of human pathogen marker genes in the vegetable phyllosphere. Values presented in the heatmap were log-transformed. The bar plot on the left indicates the total absolute abundance and detection rate of each human pathogen marker gene (HPMG). The bar plot on the top shows the total abundance of HPMGs from each vegetable species, respectively. The red text represents pathogenic protists. Marker genes of *Klebsiella pneumoniae* and *Cronobacter* spp. showed the highest abundance in the phyllosphere of Chinese chives (sign test, **p < 0.01). “OPLGs” represents organically produced leafy greens and “CPLGs” represents conventionally produced leafy greens.

Twenty-six potential human pathogens including 61 marker genes were detected in 86 of 96 vegetable phyllosphere samples (Fig. 2). *Staphylococcus aureus, Klebsiella pneumoniae, Pseudomonas aeruginosa*, and *Cronobacter* spp. were the most prevalent and abundant pathogens occurring in 58.1%, 52.3%, 31.4% and 38.4% of all samples, respectively. Absolute abundance of *K. pneumoniae phoE, S. aureus tufA, P. aeruginosa regA*, and *Cronobacter* spp. ITS genes were 1.1 × 10^3^–4.0 × 10^6^ copies/g, 1.1 × 10^3^–1.5 × 10^5^ copies/g, 1.2 × 10^3^–5.1 × 10^4^ copies/g, and 1.7 × 10^3^–3.1 × 10^6^ copies/g, respectively. Four pathogenic protists: *Acanthamoeba* spp., *Cryptosporidium, Giardia lamblia*, and *Entamoeba histolytica* accounting for 1.8 % of the overall abundance of HMPGs, were only positive in less than ten samples (Fig. 2), of which *Acanthamoeba* spp. and *Giardia lamblia* had the relatively high abundance of 1.6 × 10^2^–3.0 × 10^4^ copies/g and 3.2 × 10^2^–4.9 × 10^4^ copies/g, respectively. There were also significant differences in HPMGs detected in different vegetable species (Supplementary Fig. S7A, *P* = 0.001) but not between OPLGs and CPLGs (Supplementary Fig. S7B). The phyllosphere of Chinese chives had the greatest total abundance of HPMGs (ranging from 2.7 × 10^3^ to 4.0 × 10^6^ copies/g), followed by lettuce, cabbage, and Chinese cabbage (Fig. 2). *K. pneumoniae* and *Cronobacter* spp. ITS were significantly enriched in the phyllosphere of Chinese chives (Fig. 2, *p* < 0.01).

**Fig. 3:**
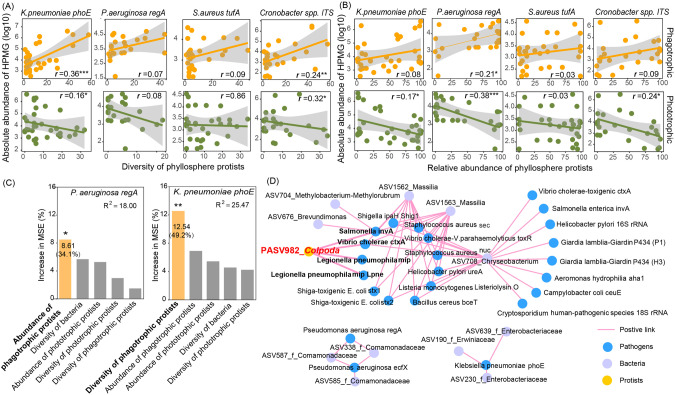
Correlations between phyllosphere protists and human pathogen marker genes. **A** Diversity and **B** relative abundance of phyllosphere protists and the absolute abundance of the most prevalent human pathogen marker genes (HPMGs) (*Staphylococcus aureus tufA*, *Pseudomonas aeruginosa regA*, *Klebsiella pneumoniae phoE*, and *Cronobacter spp. ITS*). **C** The relative importance of microbial characteristics (diversity based on Shannon index and relative abundance) in predicting the abundance of *P. aeruginosa regA* and *K. pneumoniae phoE* in the phyllosphere of leafy greens. **p < 0.01. **D** Spearman correlations between the absolute abundance of HPMGs and microbial taxa (ASV) (≥ |0.7|; *p* < 0.01) indicate that *colpoda* was the key protist closely linked to HPMGs.

The overall abundance of HPMGs was significantly and positively linked to the relative abundance (Supplementary Fig. S8A; *p* < 0.001) and diversity (Supplementary Fig. S8C; *p* < 0.05) of phagotrophic protists rather than bacteria (Supplementary Fig. S9). While the relative abundance of phototrophic protists negatively correlated with the overall abundance (Supplementary Fig. S8G; *p* < 0.05) and diversity of HPMGs (Supplementary Fig. S8H; *p* < 0.05). Specifically, the absolute abundance of Top4 prevalent HPMGs (*K. pneumoniae phoE, S. aureus tufA, P. aeruginosa regA*, and *Cronobacter* spp. ITS genes) positively correlated to the diversity and relative abundance of phagotrophic protists, but negatively correlated to the relative abundance of phototrophic protists (Fig. 3A, B) rather than bacteria (Supplementary Fig. S9). In particular, the diversity of phagotrophic protists positively correlated with the absolute abundance of *K. pneumoniae phoE* (Fig. 3A; *p* < 0.001). The absolute abundance of *P. aeruginosa regA* was significantly and positively associated with the relative abundance of phagotrophic protists (Fig. 3B; *p* < 0.05). Random forest analyses statistically supported that diversity and the relative abundance of phagotrophic protists best predicted the absolute abundance of *K. pneumoniae phoE* (*p* < 0.01), explaining more than half of the variation in the absolute abundance of *K. pneumoniae phoE*. Co-occurrence analysis identified 12 ASVs that were strongly and positively associated with HPMGs (Spearman correlation ≥0.7, *p* < 0.01; Fig. 3D). The phagotrophic protist *Colpoda* (PASV982), which acted as a connector in the network (Supplementary Fig. S10) was closely related to the HPMGs of *Salmonella, L. pneumophila* and *V. cholerae* (Fig. 3D). Bacterial ASV708 assigned to *Chryseobacterium* was most tightly linked to pathogens, with up to 13 pathogens including 18 marker genes (Fig. 3D).

### Isolated bacterial endosymbionts of phyllosphere protists

**Fig. 4:**
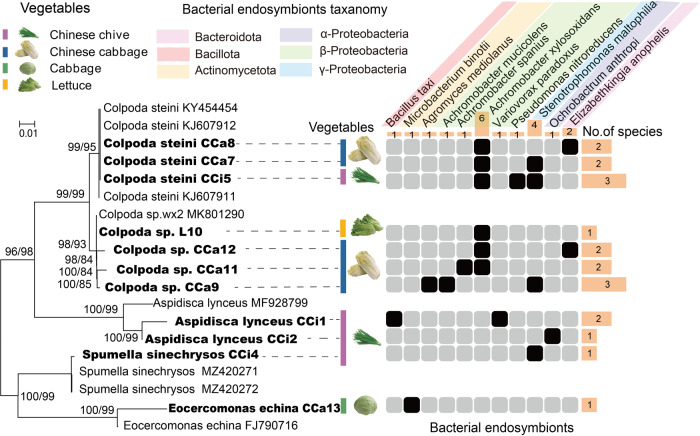
Maximum-likelihood phylogenetic trees of isolated phyllosphere protists and their bacterial endosymbionts. Sequences obtained in this study were marked in bold. Orange bars on the right and the top display the total number of endosymbionts bacteria isolated from phyllosphere protists, and the detection frequency of each endosymbiont bacteria, respectively.

**Fig. 5:**
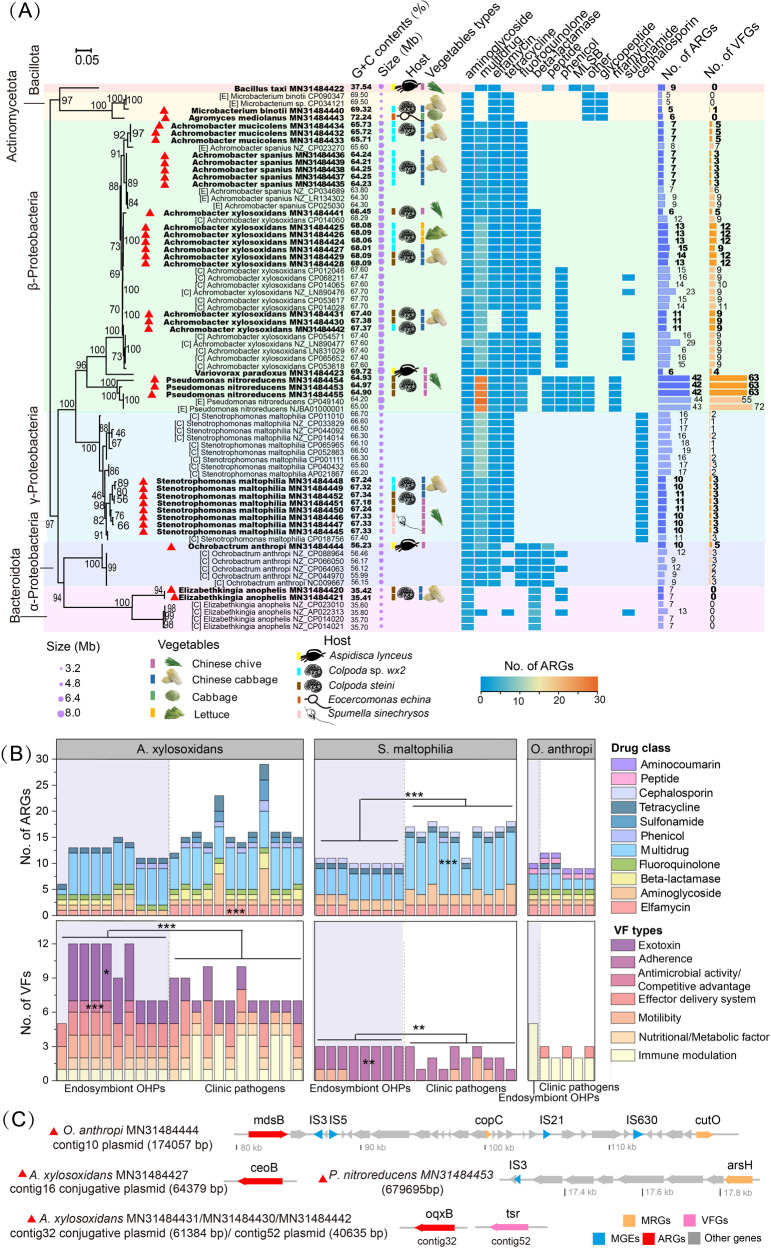
Key features of endosymbiont genomes isolated from phyllosphere protists. **A** Maximum-likelihood phylogenetic trees of endosymbiont genomes and reference genomes with 1000 trials. Sequences obtained in this study were marked in bold. Potential human opportunistic pathogens are marked with a red triangle. [E] and [C] represent the reference genomes from the environment and the clinic, respectively. The size of the purple circles indicates the genome size of each endosymbiont isolate. Heatmap denotes ARG types carried by the corresponding genomes, the purple, orange, and green bars display the total number of ARGs and VFGs carried by the corresponding isolates, respectively. **B** Comparative analysis of ARGs and VFs carried by clinical genomes and three endosymbiont genomes (*A. xylosoxidans O. anthropi* and *S. maltophilia*). **C** Schematic of the genetic organization of ARGs, MRGs, and MGEs. *p < 0.05, **p < 0.01, ***p < 0.001.

To gain deeper insight into the positive relations between phyllosphere protists and OHPs, we isolated eleven phyllosphere protists and their bacterial endosymbionts. All phyllosphere protist isolates (Supplementary Table S1) were phagotrophic protists, annotated as *Colpoda steini PAPSc-4, Aspidisca lynceus, Spumella sinechrysos, Eocercomonas echina*, and *Colpoda* sp*. wx2*. Among them, *Colpoda steini PAPSc-4* was the most prevalent protist detected from Chinese Chives, Chinese Cabbages, and lettuce (Fig. 4). Obtained bacterial endosymbionts (36) were characterized as β-proteobacteria, γ-proteobacteria, α-proteobacteria, Firmicutes, Actinobacteria, and Bacteroidetes (Figs. 4 and 5A), mostly gram-negative. On average two different bacterial endosymbionts (mainly proteobacteria) were internalized in the isolated phyllosphere protists assigned to *Colpoda steini* and *Colpoda sp*. (Fig. 4). Certain protist cultures contained endosymbiotic bacteria from different phyla. For example, *Colpoda steini CCa8* harbored endosymbionts belonging to Bacteroidetes and Proteobacteria, while isolated *Colpoda* sp. harbored endosymbionts belonging to Actinobacteria and Proteobacteria (Fig. 4). For the most part, specific bacterial taxa were internalized in respective protist hosts (in one protist host or protists from the same genus), except for *Stenotrophomonas maltophilia*, which was detected within both *Aspidisca lynceus* and *Colpoda* (Fig. 4). In addition, we compared the bacterial community based on 16S rRNA amplicon sequencing data with the isolated endosymbiont OHPs at the genus level (Supplementary Fig. S11). The genus *Achromobacter* was not observed in the free-living community, while three species of this genus, *A. xylosoxidans, A. spanius*, and *A. mucicolens* were isolated within protists. The genus *Elizabethkingia* was not found in amplicon sequencing data of Chinese cabbages, where endosymbiont *Elizabethkingia anopheles* were isolated.

A total of 419 ARGs belonging to 82 types including 11 drug classes were detected in the endosymbionts, dominated by genes conferring resistance to aminoglycosides and those encoding for multidrug efflux pumps (Fig. 5A). Each endosymbiont carried at least five ARGs, of which *rpsL* and *rpsJ* showed high detection frequency (Fig. 5A; Supplementary Fig. S12). We also identified a total of 333 MRGs associated with resistance to arsenic, iron, selenium, chromium, mercury, copper, aluminum, and multimetal in 35/36 endosymbiont isolates, of which multimetal, copper, and arsenic resistance genes were the predominant MRGs (Fig. 5A, Supplementary Fig. S13). There was a significant positive correlation between the number of ARGs and MRGs possessed by endosymbiont isolates (Supplementary Fig. S14; *p* < 0.001).

### Virulence and resistance traits of potential OHP endosymbionts

According to the A-to-Z database and previous reports, 94.4% (34/36) of endosymbionts isolated from phyllosphere protists were potential OHPs, including *Pseudomonas nitroreducens, Achromobacter xylosoxidans, Ochrobactrum anthropi*, *S. maltophilia*, *Elizabethkingia anophelis*, *Microbacterium binotii*, and *Achromobacter spanius* (Fig. 5A, Supplementary Table S3). A total of 301 VFGs across eight categories were detected in 91.2% of the OHPs. Of greatest concern, except for *E. anophelis* and *A. mediolanus*, most OHPs possessed multiple ARGs and VFGs and there was a significant positive correlation between the number of ARGs and VFGs (Supplementary Fig. S14; *p* < 0.001). *P. nitroreducens* carried a significantly high number of VFGs (63) and ARGs (42), mostly of the multidrug resistance genes, followed by *A. xylosoxidans, O. anthropi* and *S. maltophilia* (Fig. 5A; Supplementary Figs. S12 and S15). Compared with the clinical reference genomes, endosymbionts annotated as *A. xylosoxidans* and *S. maltophilia* carried low numbers of ARGs involving elfamycins and multidrug resistance genes (*p* < 0.001), respectively, but showed higher virulence potential than clinical genomes (Fig. 5B, Supplementary Fig. S16; *p* < 0.01). More specifically, *A. xylosoxidans* and *S. maltophilia* possessed more exotoxin VFs (*cyaB* gene; *p* < 0.05) as well as antimicrobial activity/competitive advantage VFs (*MtrD* gene; *p* < 0.001), and adherence VFs (Type IV pili; *p* < 0.01) than clinical genomes, respectively (Fig. 5B, Supplementary Figs. S15 and S16).

Furthermore, we analyzed the transferability potential for ARGs, MRGs, and VFGs carried by endosymbiont OHPs. Here, co-localizations of ARGs, MRGs, and MGEs were observed in both plasmids and chromosomal contigs. For example, on a plasmid contig of *O. anthropi MN31484444*, ARG *mdsB* was located near two insertion sequences (IS) *IS3* and *IS5* ( <10 kb, Fig. 5C), meanwhile, MRGs *copC* and *cutO* were located near IS fragments *IS21* and *IS630*, respectively. MRGs *arsH* was observed near to *IS3* on a chromosomal contig of *P.nitroreducens MN31484453*. Besides, *ceo*B and *oqx*B genes encoding for multidrug antibiotic efflux pumps were identified on conjugative plasmid contigs of *A. xylosoxidans* (Fig. 5C).

## Discussion

The vegetable phyllosphere is an important habitat for protists [73, 74]. Here, although phyllosphere protists were variable between replicate samples, *Colpoda* were the most dominant and prevalent phyllosphere protists. This result is consistent with previous isolation-based evidence of *Colpoda* spp. dominance on the leaves [75] and amplicon-based evidence of *Colpoda* dominance on solanaceous plants [76]. Interestingly, we observed host-specific protists and no shared protistan ASV across vegetable species, suggesting that host selection of vegetable species largely shaped the distribution of protistan communities. This is potentially associated with the sensitivity to environmental changes of protists [77], as protists are likely to colonize selectively according to leaf surface factors, such as blade roughness, stomatal density, hydrophobicity, and level of epicuticular wax [78, 79]. In this study, cabbage harbored the lowest protistan diversity possibly attributed to a high level of hydrophobic waxy cuticle that reduces water on the surface and is thus unfavorable for protist colonization [80]. The species-specific protistan could also drive through bottom-up forces of bacterial communities, which are major prey for phagotrophic protists and are significantly influenced by host selections [81]. Besides, in this study, production processes hardly influenced the alpha- or beta-diversity of phyllosphere protistan communities, which differed from soil protistans communities exhibiting distinct patterns between organic and chemically fertilized soils [82]. One explanation for this could be the multiple factors in the open nature of the phyllosphere [83], including unknown plant cultivation, transportation, and produce-packing conditions [84], which may weaken the influence of different production processes on phyllosphere protistan variations [85].

Host selection [86] and consequent different protistan communities and different microbial co-occurrence patterns may also affect the colonization of human pathogens in the phyllosphere, leading to different risks of human pathogens in vegetables. In this study, Chinese chives and lettuces might constitute a higher epidemiological risk than cabbage due to their higher content of OHPs. Specifically, *S. aureus, K. pneumoniae, P. aeruginosa*, and *Cronobacter* spp., were the prevalent OHPs found in all studied vegetable species. These results suggest that the ubiquitous detection of human pathogens in leafy greens is a vital health concern and that we should wash leafy greens repeatedly before eating them raw whether organically or conventionally grown [87]. The significant positive association between phyllosphere phagotrophic protists (both the diversity and the relative abundance) and the abundance of the Top 4 HPMGs, and the highest explanation of the phagotrophic protists on the abundance of *K. pneumoniae phoE* and *P. aeruginosa regA* both indicated a strong connection between phyllosphere phagotrophic protists and OHPs. This strong connection can be partially explained by the selective predation of phagotrophic protists on various human pathogens, including *K. pneumoniae*, *Pseudomonas aeruginosa*, and *Staphylococcus aureus* [15]. Phagotrophic protists can also influence the colonization of OHPs via regulating nutrient exchange [88] or pathogen-suppressing secondary metabolite genes [89].

Another way to link phagotrophic protists and OHPs is to become endosymbiosis. A key finding when isolating phyllosphere protists and their endosymbionts was that phagotrophic protists *Colpoda steini*, HPMGs-associated protists in the network, internalize multiple antibiotic-resistant OHPs, including *P. nitroreducens*, *A. xylosoxidans* and *S. maltophilia*. *A. xylosoxidans* and *S. maltophilia* possessing the second highest number of ARGs and VFGs in this study, are common amoeba-resisting endosymbionts [90–92] associated with cystic fibrosis and respiratory infections [93, 94]. *P. nitroreducens*, never been reported as an endosymbiont of protists before, carried the highest number of ARGs and VFGs and has a close phylogenetic relationship with common protists endosymbiont *P. aeruginosa* [34, 95]. Hence, similar to *P. nitroreducens*, *P. aeruginosa* may establish a positive correlation with the relative abundance of phyllosphere phagotrophic protists through endosymbiosis, though we did not isolate it. In addition, four OHPs species only found within protists indicated that protists as a potential reservoir for OHP may transfer endosymbiont OHPs from other environments, resulting in the potential increase of OHPs in the phyllosphere. Moreover, all isolated protist-adapted endosymbiont OHPs have the potential to return to the environment even with more environmental persistence [96]. This may partly explain why some endosymbiont OHPs occurred in both free-living and isolated communities. Nonetheless, the findings of this study did not reveal the percentage of OHPs released from protists to the environment and the relative contribution of protistan predation on the variation of OHPs in the phyllosphere, which calls for more future research to be addressed. Given the limited culturable phyllosphere protists and the absence of obligate intracellular bacteria that cannot be cultivated without their hosts in this study, there may be more pathogenic endosymbionts OHPs within protists, indicating that more attention should be directed to the role of phagotrophic protists as important vectors of the transmission of OHPs in the vegetable phyllosphere.

Two main survival strategies of intracellular bacteria have been reported for digestion resistance [97]: escaping into the cytosol [98]; subverting antimicrobial mechanisms including removing toxic compounds, preventing phagosome-lysosome fusion, modulating phagosomal pH, etc [19]. In this study, the specific resistance and virulence traits of OHPs within protists may act as subvert antimicrobial mechanisms partially contributing to resisting digestion and facilitating the survival of the endosymbionts [97]. For instance, efflux pumps SmeDEF (ARGs) carried by OHPs *S. maltoph*ilia have been demonstrated to be induced by secondary metabolites of amoeba to directly expel toxic compounds secreted by *Acanthamoeba castellanii* [11], allowing the survival and intracellular growth of S. maltophilia, and contribute to intrinsic multidrug resistance of *S. maltophilia* [99]. The VF T6SS such as VgrG-1 found in *P. nitroreducens* were reported to defend against protist digestion by injecting antimicrobial toxins [100–102], or causing cytotoxic actin-crosslinking in the predatory amoeba *Dictyostelium discoideum* [103]. As for MRGs, arsenic (such as arsenic resistance operon *arsH*) and copper resistance genes (such as *copA*) carried by *A.xylosoxidans* may help adapt to copper and arsenic poisoning by *Dictyostelium* grazing and thus escape phagocytosis [17].

In our assay, most isolated endosymbionts OHPs carried both VFGs, MRGs, and ARGs, and the number of VFGs or MRGs and ARGs was positively correlated, indicating the co-occurrence profiles among them. The intimate co-occurrence and the high cotransfer potential among ARGs, VFGs, and MRGs have more often observed in genomes from pathogen species [104], such as *K. pneumonia*e [105] and enterococci [106], which potentially complicate the treatment of OHPs infections. Here, the residency of VFGs, MRGs, and ARGs on MGEs (plasmid fragments) from OHPs further indicated the transmission potential of virulence and resistance properties using mobile genetic elements via HGT. Moreover, HGT has been reported to increase greatly within phagotrophic protists contributing to the adaptation and evolution of endosymbionts OHPs [107]. For example, the conjugation frequency of E. coli strains engulfed by the ciliate increased 2000- to 4000-fold after a full digestion cycle [108]. Similarly, protozoal predation enhances the transformation of a gene cassette in *Vibrio cholerae* by as much as 405-fold through SOS-regulated DNA integration [109]. These indicated that the HGT of ARGs, VFGs, and MRGs among endosymbiont OHPs may be further expedited under the predation pressure of protists [110], and thus contribute to the survival of more endosymbionts [111] and the emergence of resistant OHPs. In addition, endosymbionts *A. xylosoxidans* and *S. maltophilia* showed higher virulence potential including invasive adenylate cyclase /haemolysin, Resistance/nodulation/division (RND)-type efflux pump, attachment to host cells and biofilm formation, but lower antibiotic resistance potential than clinical genomes, which probably relate to HGT and less frequent exposure to antibiotics [105]. Overall, phyllosphere protists can be important vectors for the transmission of resistant OHPs and the resistance and virulence traits of OHPs may even be exchanged among endosymbionts OHPs which needs more attention.

## Conclusions

We revealed that phagotrophic protists in the phyllosphere of leafy greens can harbor multiple resistant OHPs and are closely related to OHP abundance. In these leafy greens, *Colpoda* was identified as the key phagotrophic protist of OHPs. ARGs, VFGs, and MRGs co-occur at high frequencies in endosymbionts OHPs with the potential for HGT, which contributes to the emergence of resistant OHPs in the phyllosphere of leafy greens and poses risks to consumers, and within the one health perspective, to the environment. Besides, host selection of vegetable species largely affects the distribution of OHPs and protistan communities. This work fills the knowledge gap regarding the association between phyllosphere protists and HPMGs and emphasizes that phyllosphere phagotrophic protists in leafy greens are more important in the transmission and evolution of OHPs than hitherto assumed. These findings advance our understanding of phyllosphere protists and are valuable for environmental health and food safety under the One Health framework.

### Supplementary information

Supplemental material

## Data Availability

All raw sequencing data were deposited to the Sequence Read Archive (SRA) under the number PRJNA855267.
