# Supplementary material for: Phagotrophic protists preserve antibiotic-resistant opportunistic human pathogens in the vegetable phyllosphere
Source: ISME Commun. 2023 Sep 2;3:94. doi: 10.1038/s43705-023-00302-z (PMC10475086; doi:10.1038/s43705-023-00302-z)
Supplement: Supplementary file 1 — Supplemental material [file 43705_2023_302_MOESM1_ESM.docx]

**Supporting Information**

**Phagotrophic protists preserve antimicrobial-resistant opportunistic human pathogens in vegetable phyllosphere**

Chenshuo Lin^1, 2^, Li-Juan Li^1, 2^, Kexin Ren^1^, Shu-Yi-Dan Zhou^3^, Alain Isabwe^1^, Le-Yang Yang^1, 2^, Roy Neilson^4^, Xiao-Ru Yang^1^, Eddie Cytryn^5^, Yong-Guan Zhu^1, 6^*

^1^ Key Laboratory of Urban Environment and Health, Institute of Urban Environment, Chinese Academy of Sciences, 1799 Jimei Road, Xiamen 361021, China

^2^ University of Chinese Academy of Sciences, 19A Yuquan Road, Beijing 100049, China

^3^ Key Laboratory of Vegetation Restoration and Management of Degraded Ecosystems, South China Botanical Garden, Chinese Academy of Sciences, Xingke Road 723, Tianhe District, Guangzhou, 510650, China

^4^ Ecological Sciences, The James Hutton Institute, Dundee, DD2 5DA, Scotland, UK

^5^ Department of Soil Chemistry, Plant Nutrition and Microbiology, Institute of Soil, Water and Environmental Sciences, The Volcani Institute, Agriculture Research Organization, 7528809 Rishon Lezion, Israel

^6^ State Key Laboratory of Urban and Regional Ecology, Research Center for Eco-Environmental Sciences, Chinese Academy of Sciences, Beijing 100085, China

***Author for correspondence:**

Yong-Guan Zhu (ygzhu@rcees.ac.cn) Phone: 86-592-6190997

**Supplementary Methods**

**Methods S1**

PCR of eukaryotic 18S rRNA gene and bacterial 16S rRNA gene were performed in a 20 μL volume consisting of 4 μL of 5× reaction buffer, 2 μL dNTPs (2.5 mM), 0.8 μL of each primer (5 μM), Fast Pfu Polymerase 0.4 μL, 2 μL Bovine Serum Albumin (2mg/mL BSA), 10 ng of DNA template, and the rest being ddH_2_O. Thermal cycling for eukaryotic 18S rRNA gene was carried out with an initial denaturation step at 95°C for 3 min followed by 37 cycles of initial denaturation at 95°C for 30 s, annealing at 53°C for 30 s, extension at 72°C for 45 s and a final elongation step at 72°C for 10 min. Amplification of 16S rRNA gene was performed with the following temperature regime: 95°C for 3 min followed by 27 cycles for the first set of primers, 13 cycles for the second set of primers of 95°C for 30 s; 55°C and for 30 s; 72°C for 45 s, and a final extension step of 72°C for 10 min. The quality of PCR amplification was verified by gel electrophoresis (2% agarose).

**Methods S2**

This included TaqMan Gene Expression Master Mix (Applied Biosystems, USA), bovine serum ampere (1 mg/mL, Sigma, USA), forward and reverse primers (0.9 μM/L), probe (0.25 μM/L), DNA (5 ng/μL) and nuclease-free water mixed in a final volume of 100 µL per reaction. Thermal cycling was carried out with an initial denaturation step at 50 °C for 2 min,95 °C for 10 min followed by 40 cycles of 95°C for 15 s and 60°C for 1min. A ten-fold serially diluted standard plasmid carrying each targeted gene was constructed to establish standard curves. The amplification efficiency of these pathogenic marker genes was in the range from 90.67% to 109.13% (Table S2). A gene detected in three technical replicates was considered positive.

**Supplementary Figures**

**
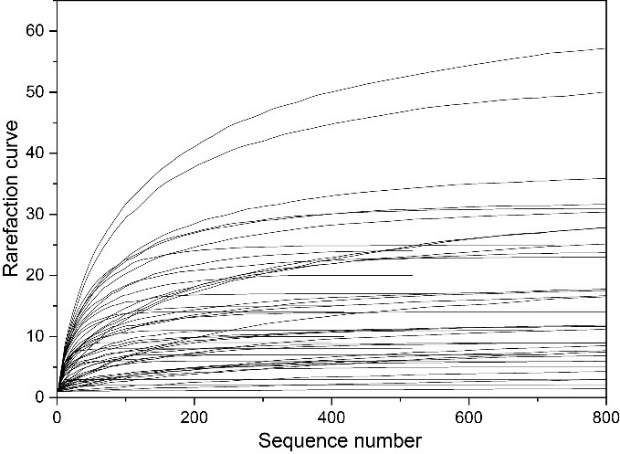
**

**Fig. S1** Rarefaction curve of protistan ASVs in the vegetable phyllosphere

**
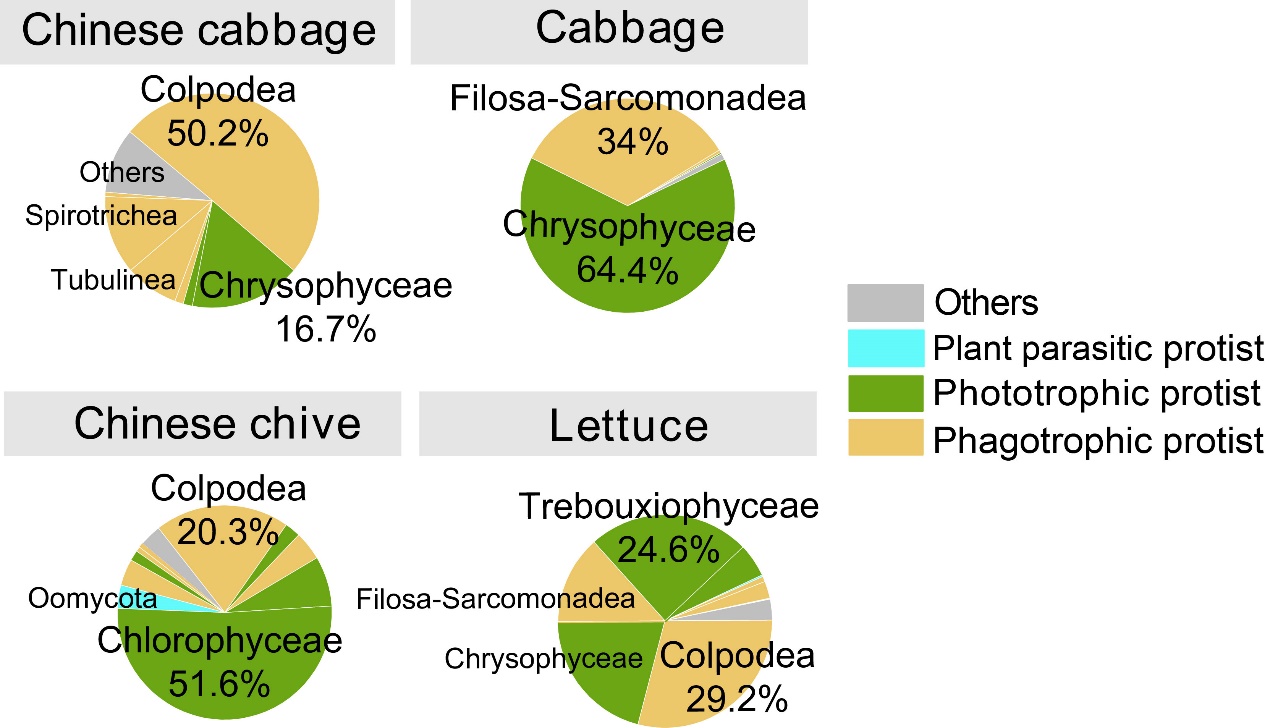
**

**Fig. S2. Main phototrophic and phagotrophic protistan classes in the phyllosphere (only show relative abundance > 10%)**

**
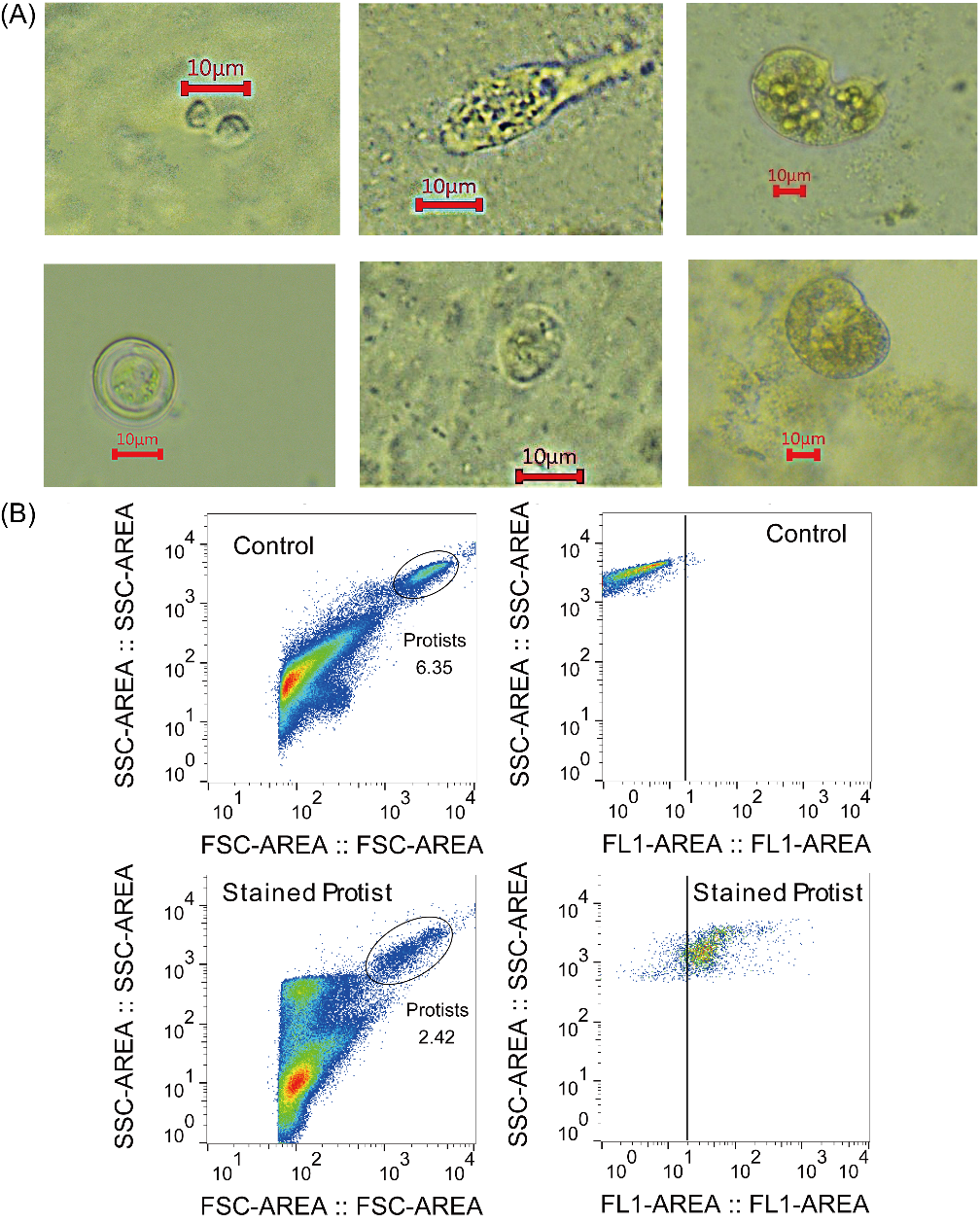
**

**Fig. S3** Micrographs of isolated phyllosphere protists from the leafy greens (A) and flow cytometry signatures of stained protists (**B**). SSC is side scatter; FSC is forward scatter.

**
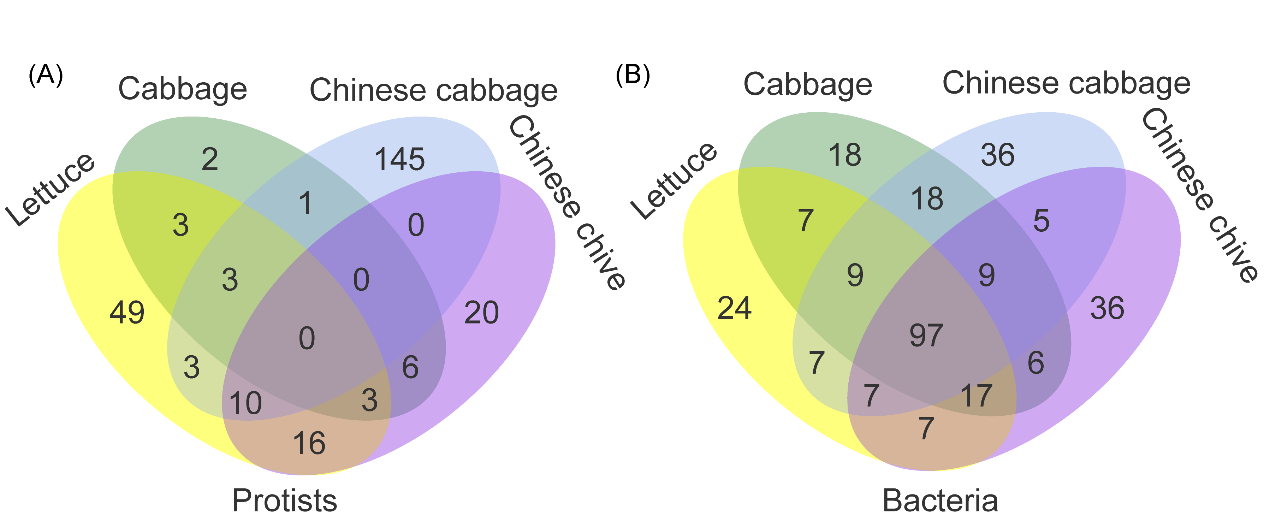
**

**Fig.** **S4** Venn diagram at protistan ASV level comparing the unique protistan (A) and bacterial (B) ASVs from four vegetable species.

**
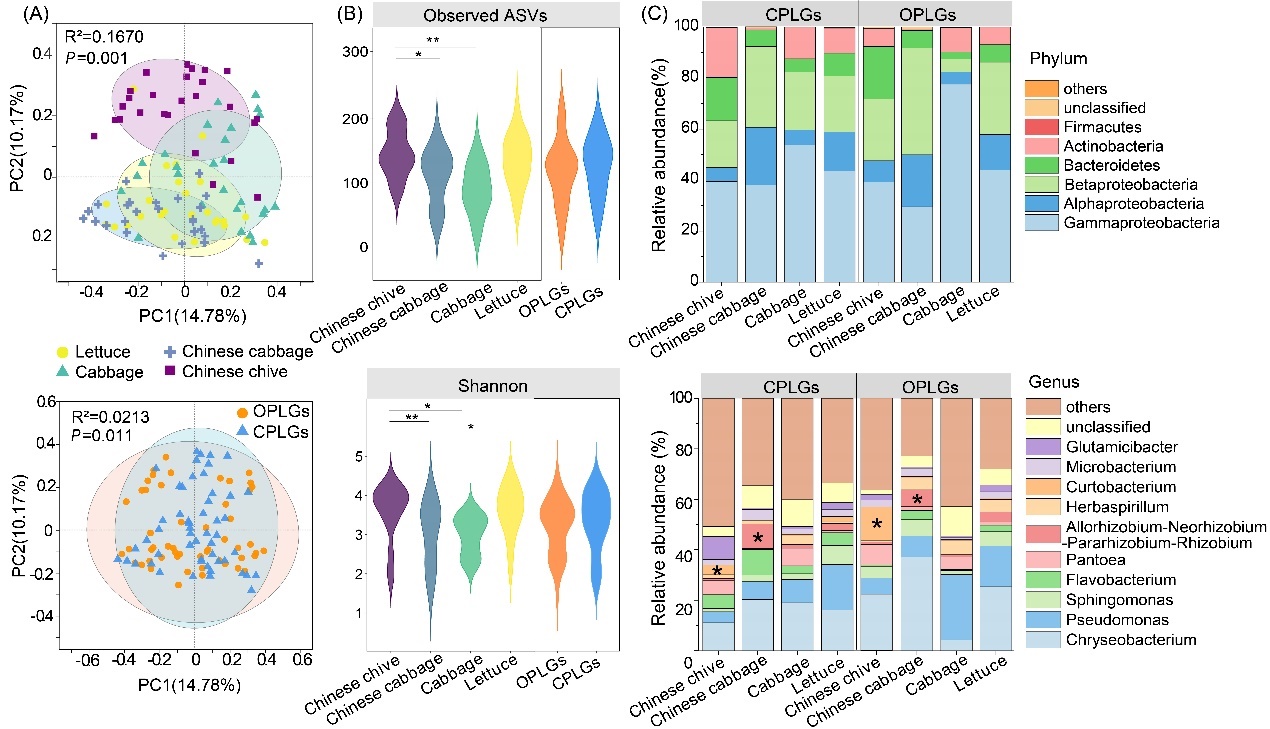
**

**Fig.** **S5** PCoA (A), diversity index (B), and composition (C) of bacterial communities in the vegetable phyllosphere across vegetable species and between production system (OPLGs and CPLGs). Significant differences of bacterial taxa and bacterial diversity across vegetable species are indicated with asterisks (sign test, **p* < 0.05, ** *p* < 0.01)


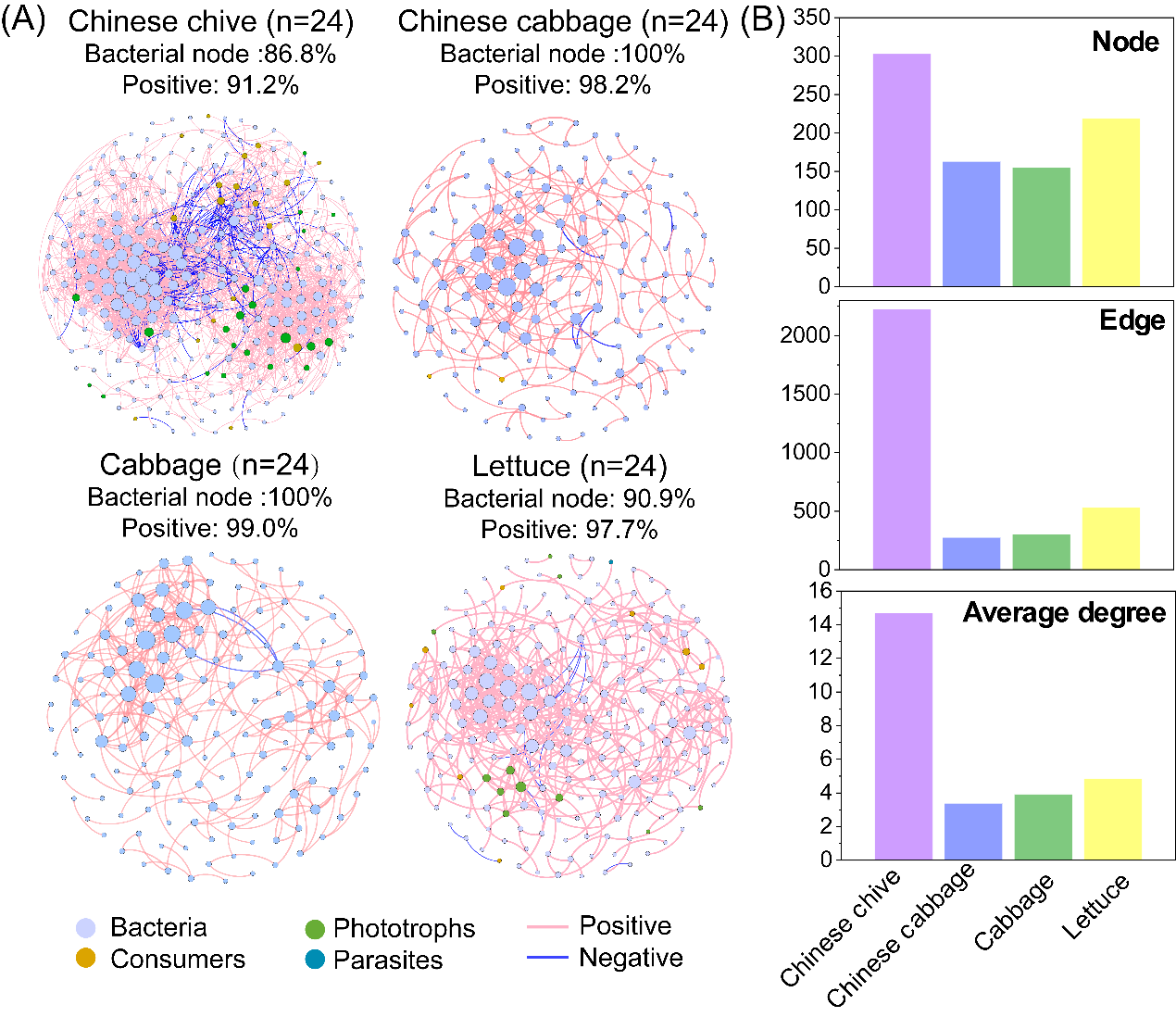


**Fig.** **S6** Co-occurrence networks constructed four studied vegetable species **(A)** and their topological properties (number of node and edge, and average degree) **(B)**. Node size is proportional to the degree of each node.


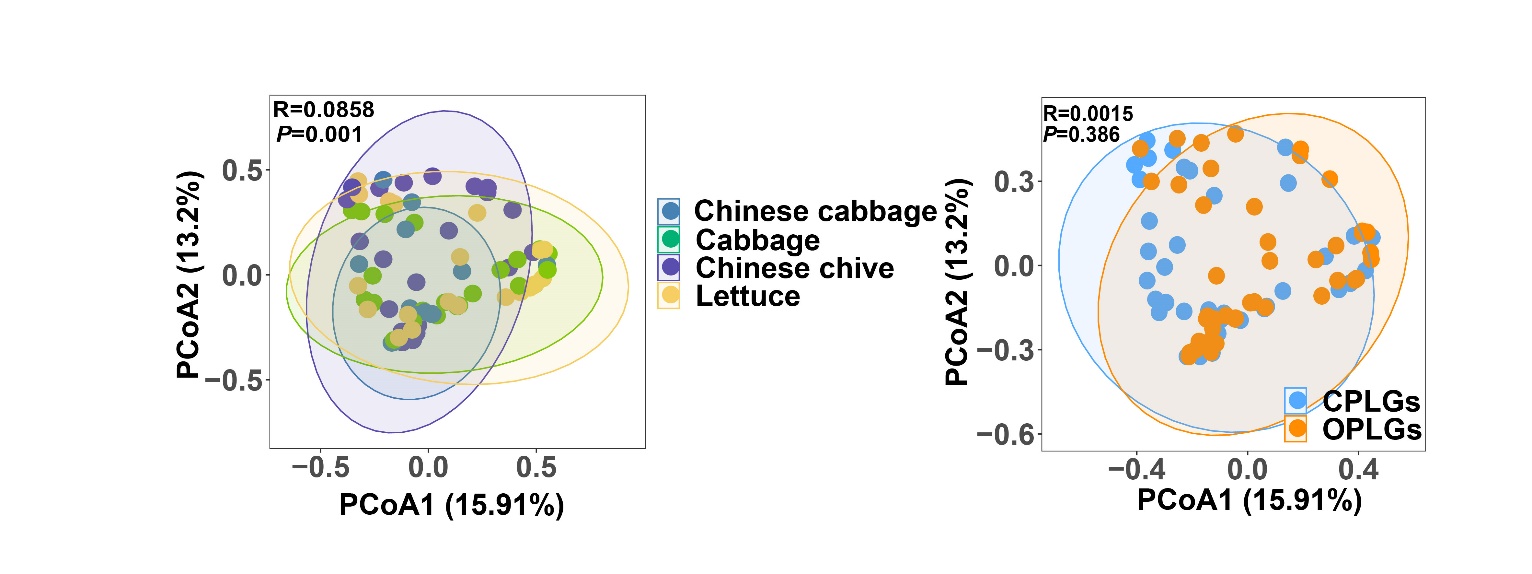


**Fig.** **S7** Principal coordinate analysis (PCoA) of human pathogen marker genes in the phyllosphere across vegetable species and between production system.


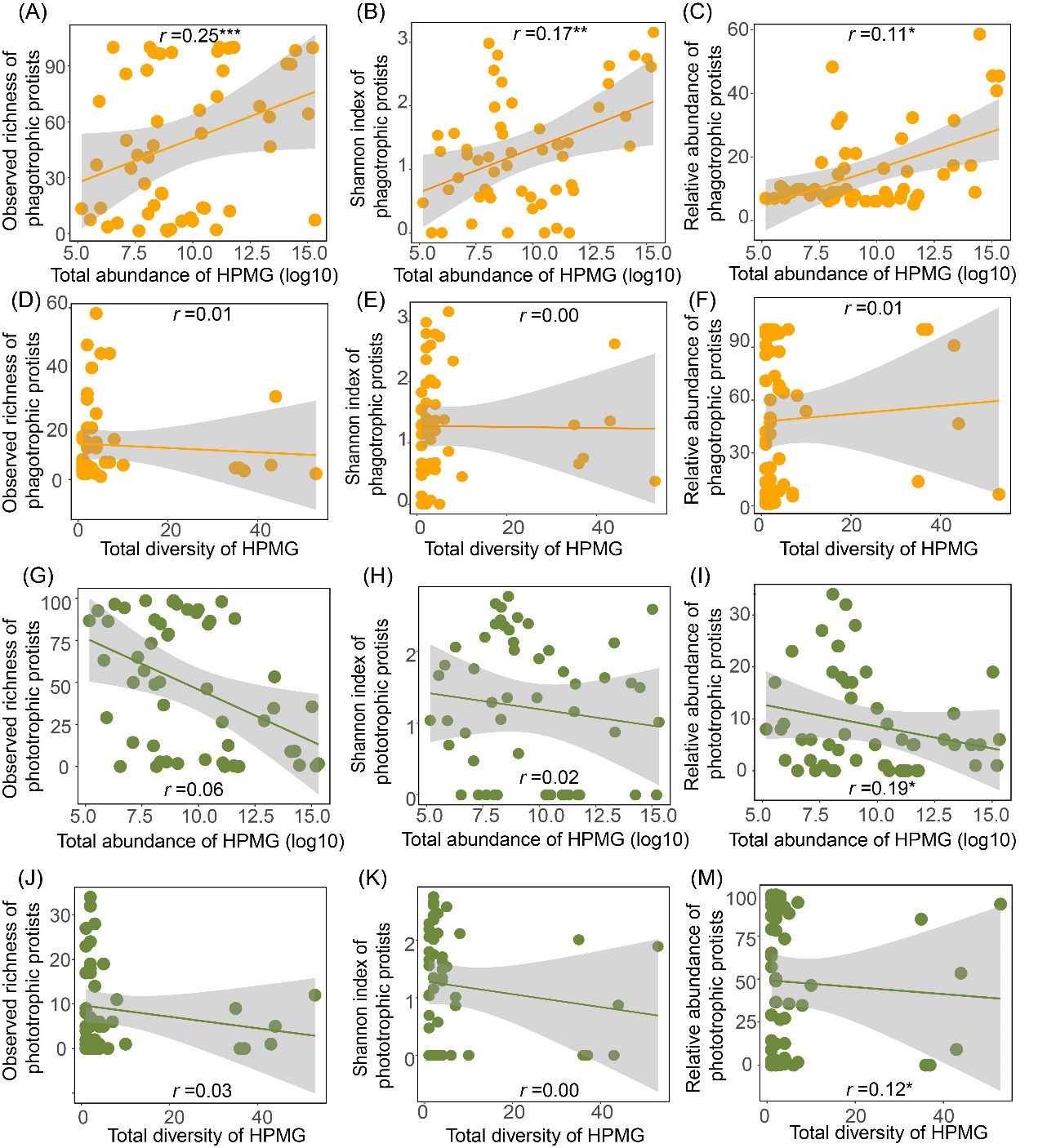


**Fig.** **S8** Correlations between the diversity (observed richness and Shannon index) and relative abundance of phyllosphere phagotrophic/phototrophic protists and the overall abundance and diversity of HPMGs (Sign test, * *p* < 0.05, ** *p* < 0.01, *** *p* < 0.001).


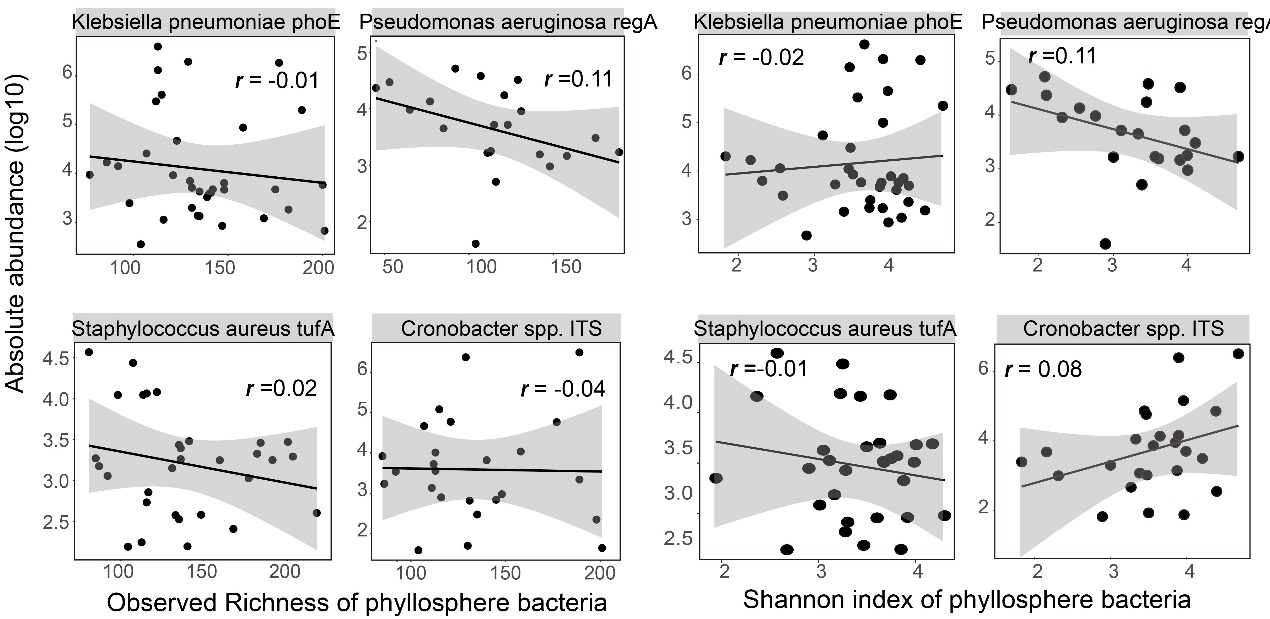


Fig. S9 Correlations between the diversity of phyllosphere bacteria (observed richness and Shannon index) and the absolute abundance of the most prevalent human pathogen marker genes (*Staphylococcus aureus tufA*, *Pseudomonas aeruginosa regA*, *Klebsiella pneumoniae phoE*, and *Cronobacter* spp. *ITS*).

**
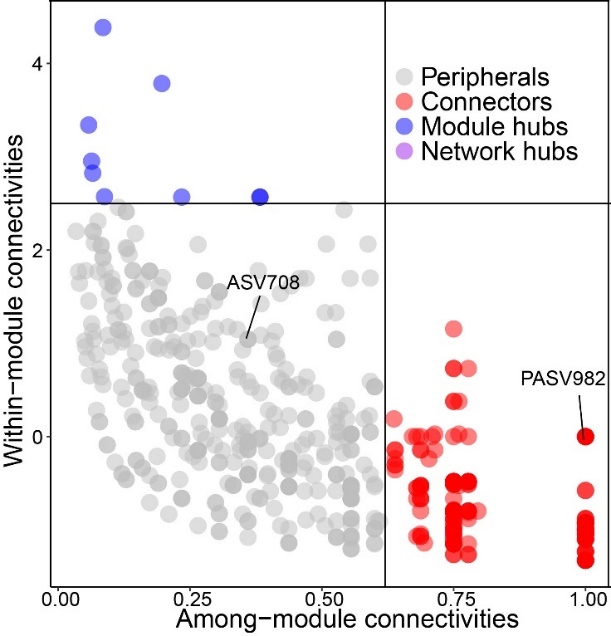
**

**Fig. S10** Distribution of bacterial and protistan ASVs based on their network roles. Nodes in the network are classified as peripherals, module hubs, and connectors depending on their roles in the network. Two marked ASVs were the protistan and bacterial nodes that most tightly connected to the HPGs.


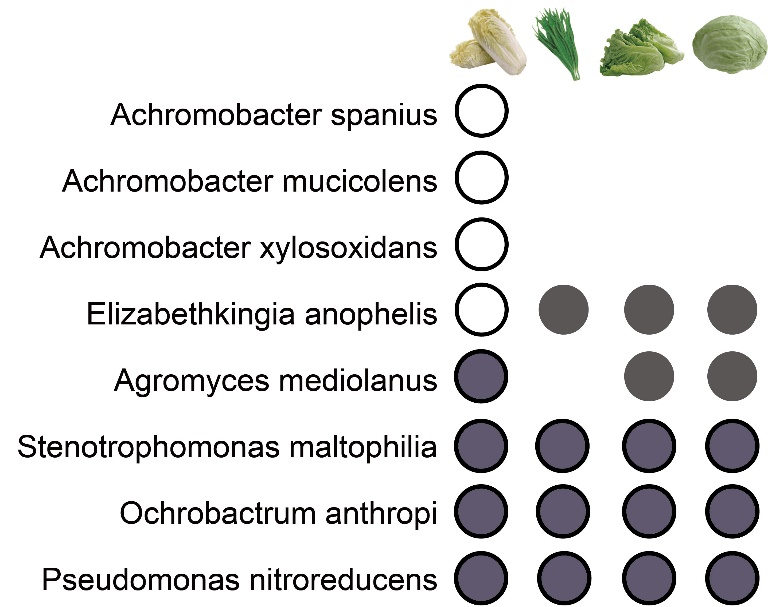


Fig. S11 Comparative analysis of the presence of the potential human pathogens (OHPs) in protistan endosymbiont and free-living communities (at genus level based on amplicon sequencing data) among different vegetable species. The black bordered hollow circle, the borderless solid circle and the black bordered solid circle represent OHPs presenting only in protistan endosymbiont community, only in free-living community, and both in protistan endosymbiont and free-living communities, respectively.


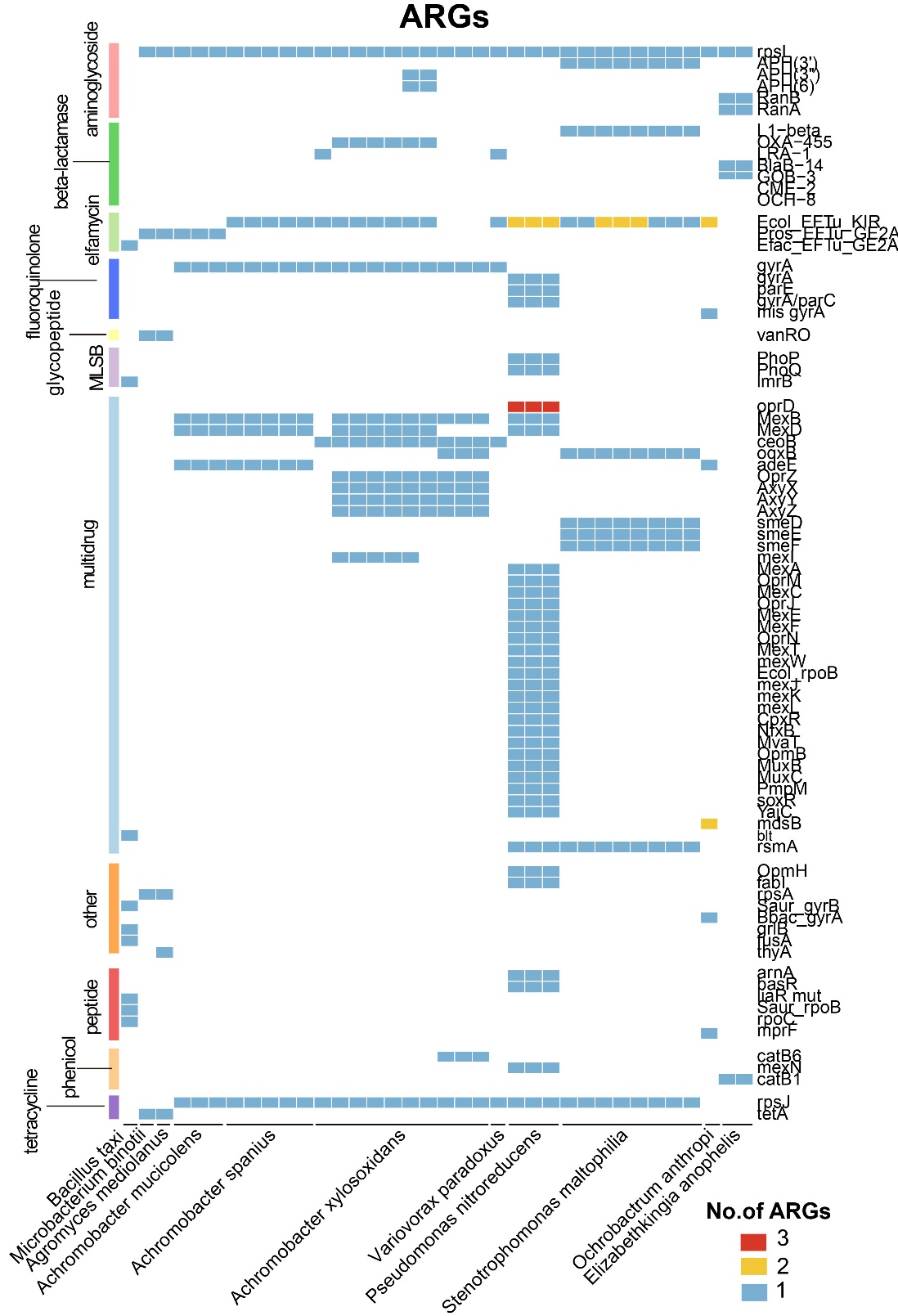


**Fig. S12** Heatmap presenting ARGs per genome for bacterial endosymbionts isolates from phyllosphere protists.

**
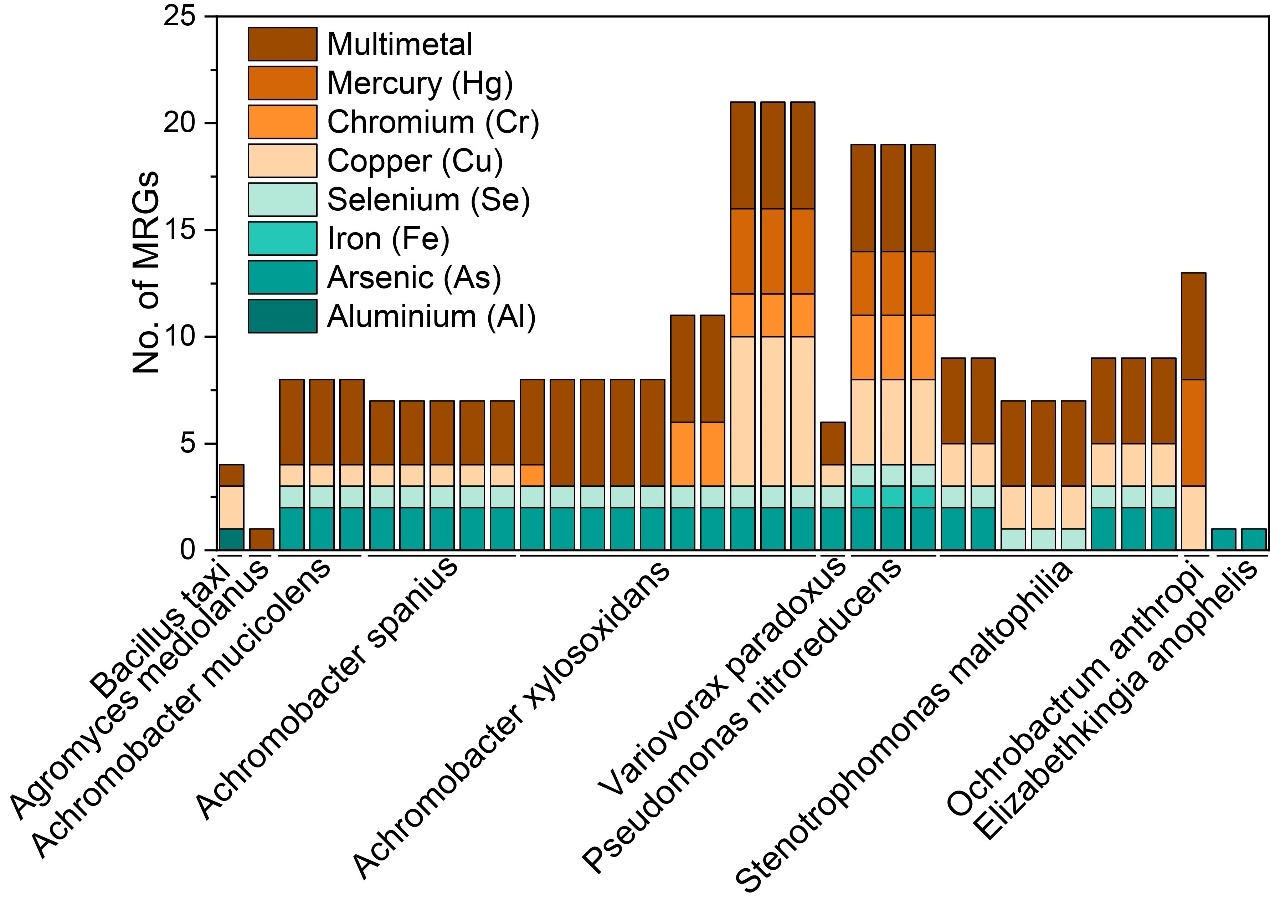
 Fig. S13** Distribution and number of MRGs in bacterial endosymbionts isolates from phyllosphere protists.

**
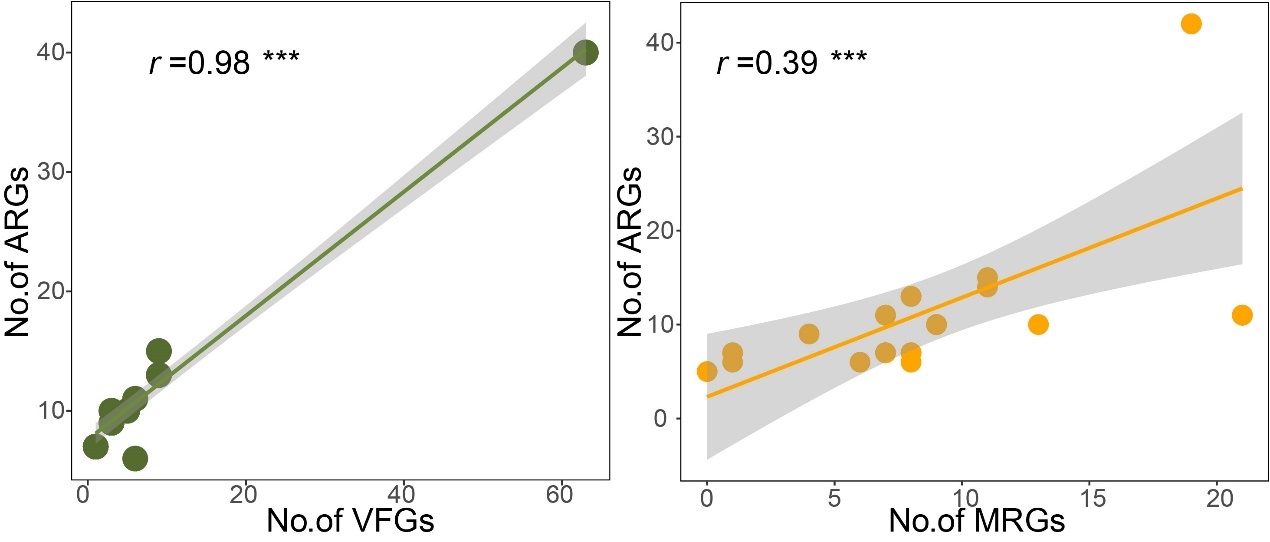
**

**Fig. S14** Correlation between the number of ARGs and the number of VFGs and MRGs carried by intercellular bacteria isolates. *** *p* < 0.001

**
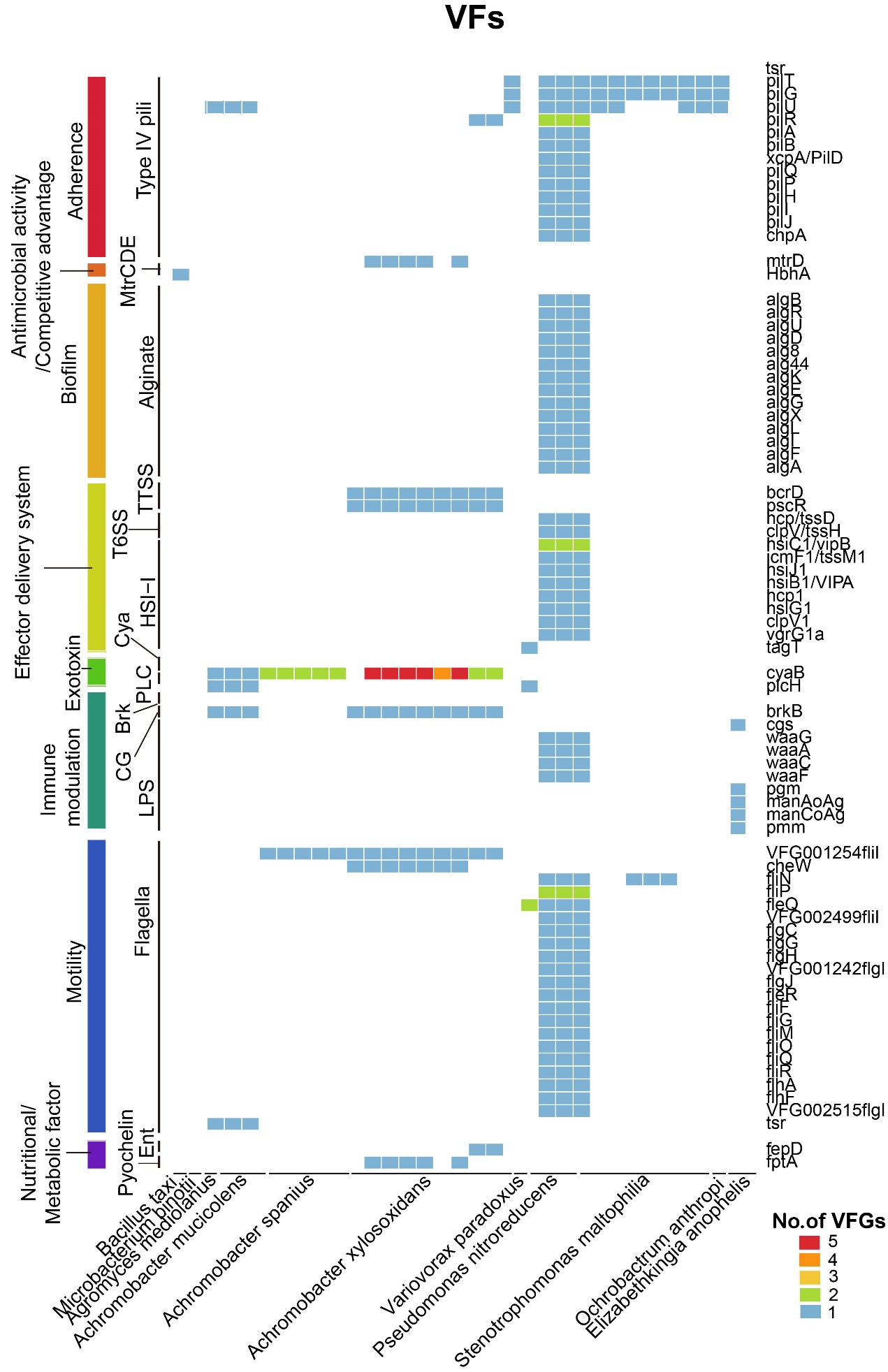
**

**Fig. S15** Heatmap presenting VFGs per genome for bacterial endosymbionts isolates from phyllosphere protists.


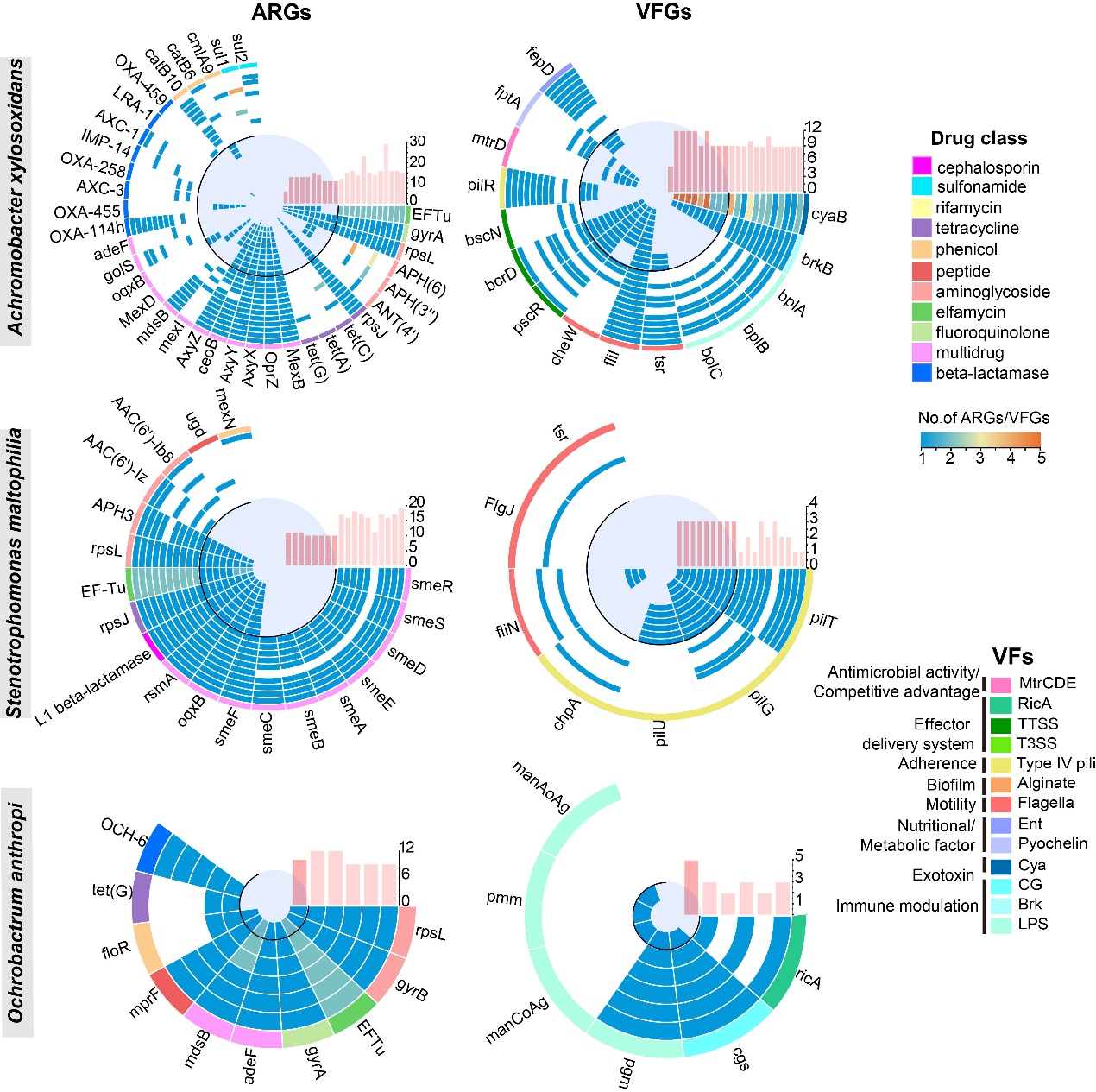


**Fig. S16** Comparative analysis of ARGs and VFGs carried by clinical genomes and three endosymbiont genomes (*A. xylosoxidans O. anthropi* and *S. maltophilia*). The bar charts show the total number of ARGs and VFGs. Genome information of endosymbionts in this study is visualized in the dark red bars with a blue background, while reference clinical genomes show in light red bars with a blank background.

**Supplementary Tables**

**Table S1**. Phyllosphere protists isolated from leafy greens that can be eaten raw

| Name | Taxonomy | Vegetable host |
| --- | --- | --- |
| *Aspidisca lynceus* *CCi1* | Alveolata; Ciliophora; Spirotrichea; Hypotrichia; Euplotida; *Aspidiscidae*; *Aspidisca lynceus* | Organic Chinese chive |
| *Aspidisca lynceus* *CCi2* | Alveolata; Ciliophora; Spirotrichea; Hypotrichia; Euplotida; *Aspidiscidae*; *Aspidisca lynceus* | Organic Chinese chive |
| *Spumella sinechrysos CCi4* | Stramenopiles; Ochrophyta; Chrysophyceae; Chromulinales; *Chromulinaceae*; *Spumella sinechrysos* | Ordinary Chinese chive |
| *Colpoda steini CCi5* | Alveolata; Ciliophora; Intramacronucleata; Colpodea; Colpodida; *Colpodidae*; *Colpoda steini* | Ordinary Chinese chive |
| *Colpoda steini CCa7* | Stramenopiles; Ochrophyta; Chrysophyceae;Chromulinales; *Chromulinaceae;* *Colpoda steini* | Organic Chinese cabbage |
| *Colpoda steini CCa8* | Alveolata; Ciliophora; Intramacronucleata; Colpodea; Colpodida; *Colpodidae*; *Colpoda steini* | Organic Chinese cabbage |
| *Colpoda sp. wx2 CCa9* | Alveolata; Ciliophora; Intramacronucleata; Colpodea; Colpodida; *Colpodidae*; *Colpoda sp. wx2* | Organic Chinese cabbage |
| *Colpoda sp. L10* | Alveolata; Ciliophora; Intramacronucleata; Colpodea; Colpodida; *Colpodidae; Colpoda sp. wx2* | Ordinary Lettuce |
| *Colpoda sp. CCa11* | Alveolata; Ciliophora; Intramacronucleata; Colpodea; Colpodida; *Colpodidae; Colpoda sp. wx2* | Ordinary Chinese cabbage |
| *Colpoda sp. CCa12* | Alveolata; Ciliophora; Intramacronucleata; Colpodea; Colpodida; *Colpodidae; Colpoda sp. wx2* | Ordinary Chinese cabbage |
| *Eocercomonas echina CCa*13 | Rhizaria; Cercozoa; Cercomonadida; Cercomonadidae; *Eocercomonas echina* | Organic Cabbage |

**Table S2**. Characterization of genome of bacterial endosymbionts strains of phyllosphere protists in this study.

| Stains | Genome Size (Mb) | G + C content (%) | Scaffolds N50 (bp) | No. of scaffolds | Reference |
| --- | --- | --- | --- | --- | --- |
| *Elizabethkingia anophelis MN31484420* | 4.09 | 35.42 | 451902 | 23 | [1] |
| *Elizabethkingia anophelis MN31484421* | 4.09 | 35.41 | 451700 | 22 | [1] |
| *Bacillus taxi MN31484422* | 5.55 | 37.54 | 287181 | 57 |  |
| *Variovorax aradoxus MN31484423* | 8.07 | 69.72 | 47752 | 322 |  |
| *Achromobacter xylosoxidans MN31484424* | 6.93 | 68.06 | 103077 | 148 | [2] |
| *Achromobacter xylosoxidans MN31484425* | 6.93 | 68.08 | 182024 | 80 | [2] |
| *Achromobacter xylosoxidans MN31484426* | 6.93 | 68.09 | 277025 | 51 | [2] |
| *Achromobacter xylosoxidans MN31484427* | 6.88 | 68.01 | 30904 | 430 | [2] |
| *Achromobacter xylosoxidans MN31484429* | 6.87 | 68.09 | 164568 | 92 | [2] |
| *Achromobacter xylosoxidans MN31484428* | 6.87 | 68.09 | 98105 | 120 | [2] |
| *Achromobacter xylosoxidans MN31484430* | 7.02 | 67.38 | 69547 | 210 | [2] |
| *Achromobacter xylosoxidans MN31484431* | 7.02 | 67.4 | 151049 | 109 | [2] |
| *Achromobacter xylosoxidans MN31484441* | 6.49 | 66.45 | 97547 | 120 | [2] |
| *Achromobacter xylosoxidans MN31484442* | 7.09 | 67.37 | 109271 | 150 | [2] |
| *Achromobacter mucicolens MN31484432* | 6.05 | 65.72 | 128871 | 92 | [3] |
| *Achromobacter mucicolens MN31484433* | 6.06 | 65.71 | 180353 | 72 | [3] |
| *Achromobacter mucicolens MN31484434* | 6.05 | 65.73 | 180287 | 61 | [3] |
| *Achromobacter spanius MN31484435* | 6.54 | 64.23 | 1370504 | 31 | [4] |
| *Achromobacterspanius MN31484436* | 6.52 | 64.24 | 1369807 | 18 | [4] |
| *Achromobacter spanius MN31484438* | 6.52 | 64.25 | 645122 | 27 | [4] |
| *Achromobacter spanius. MN31484437* | 6.52 | 64.25 | 622416 | 26 | [4] |
| *Achromobacter spanius MN31484439* | 6.54 | 64.21 | 914519 | 29 | [4] |
| *Microbacterium binotil MN31484440* | 3.25 | 69.32 | 478873 | 21 | [5] |
| *Agromyces mediolanus MN31484443* | 3.86 | 72.24 | 55404 | 121 | [6] |
| *Ochrobactrum anthropi MN31484444* | 5.08 | 56.23 | 472217 | 41 | [7] |
| *Stenotrophomonas maltophilia MN31484446* | 4.63 | 67.33 | 150656 | 50 | [8] |
| *Stenotrophomonas maltophilia MN31484447* | 4.63 | 67.33 | 232491 | 47 | [8] |
| *Stenotrophomonas maltophilia MN31484445* | 4.63 | 67.33 | 232499 | 45 | [8] |
| *Stenotrophomonas maltophilia MN31484449* | 4.63 | 67.32 | 158986 | 52 | [8] |
| *Stenotrophomonas maltophilia MN31484452* | 4.62 | 67.34 | 143810 | 56 | [8] |
| *Stenotrophomonas maltophilia MN31484448* | 4.67 | 67.24 | 145235 | 49 | [8] |
| *Stenotrophomonas maltophilia MN31484451* | 4.69 | 67.18 | 109702 | 70 | [8] |
| *Stenotrophomonas maltophilia MN31484450* | 4.67 | 67.24 | 191373 | 47 | [8] |
| *Pseudomonas nitroreducens MN31484453* | 6.48 | 64.97 | 638243 | 36 | [9] |
| *Pseudomonas nitroreducens MN31484455* | 6.51 | 64.9 | 638243 | 59 | [9] |
| *Pseudomonas nitroreducens MN31484454* | 6.48 | 64.93 | 638243 | 37 | [9] |

**Table S3**. Summary of pathogen marker genes, primers and probes used for HT-qPCR assays

| Pathogens | Marker genes | Functions | Primes and probes (5'-3', F-forward primer; R-reverse primer; P-TaqMan probe) | References ^e^ |
| --- | --- | --- | --- | --- |
| *Campylobacter jejuni/ C. coli* | *cadF* | fibronectin-binding protein | F, CTGCTAAACCATAGAAATAAAATTTCTCAC；  R, CTTTGAAGGTAATTTAGATATGGATAATCG  P, FAM-CATTTTGACGATTTTTGGCTTGA-MGB-NFQ | [10] |
| *Campylobacter jejuni* | *mapA* | membrane-associated protein | F, CTGGTGGTTTTGAAGCAAAGATT；  R, CAATACCAGTGTCTAAAGTGCGTTTAT  P, FAM-TTGAATTCCAACATCGCTAATGTATAAAAGCCCTTT-BHQ1 | [11] |
|  | *hipO* | hippuricase | F, TCCAAAATCCTCACTTGCCATT；  R, TGCACCAGTGACTATGAATAACGA  P, FAM-TTGCAACCTCACTAGCAAAATCCACAGCT-BHQ1 | [12] |
| *Campylobacter coli* | *ceuE* | periplasmic substrate binding protein | F, AAGCTCTTATTGTTCTAACCAATTCTAACA；  R, TCATCCACAGCATTGATTCCTAA  P, FAM-TTGGACCTCAATCTCGCTTTGGAATCATT-BHQ1 | [11] |
|  | *cdtA* | cytolethal distending toxin | F, TGTCAAACAAAAAACACCAAGCTT；  R, CCTTTGACGGCATTATCTCCTT  P, FAM-AAAATTTCCCGCCATACCACTTGTCCC-BHQ1 | [12] |
| *Campylobacter lari* | *pepT* | peptidase | F, TTAGATTGTTGTGAAATAGGCGAGTT；  R, TGAGCTGATTTGCCTATAAATTCG  P, FAM-TGAAAATTGGAACGCAGGTG-MGB-NFQ | [12] |
| *Vibrio cholera*/*V. parahaemolyticus* | *toxR* | toxin | F, GTTTGGCGAGAGCAAGGTTT；  R, TCTCTTCTTCAACCGTTTCCA  P, FAM-CGCAGAGTCGAAATGGCTTGG-MGB-NFQ | [10] |
| *Vibrio cholerae* | *ctxA* | cholera toxin A | F, TTTGTTAGGCACGATGATGGAT；  R, ACCAGACAATATAGTTTGACCCACTAAG  P, FAM-TGTTTCCACCTCAATTAGTTTGAGAAGTGCCC-BHQ1 | [13] |
| total *Vibrio cholerae* | *ompW* | outer membrane protein W | F1, AAGCTCCGCTCCTGTATTTGC；  F2, ACTAGCCGCTCCTGTATTTGC  R, GCTATTAACTGCCAACTCACTTTGAG；  P, FAM-CACCAAGAAGGTGACTTT-MGB-NFQ | [14] |
| Toxigenic *Vibrio cholerae* | *ctxA* | cholera toxin A | F, GCATAGAGCTTGGAGGGAAGAG；  R, CATCGATGATCTTGGAGCATTC  P, FAM-CATCATGCACCGCCG-MGB-NFQ | [14] |
| *Clostridium perfringens* | *cpe* | enterotoxin | F, AACTATAGGAGAACAAAATACAATAG；  R, TGCATAAACCTTATAATATACATATTC  P, FAM-TCTGTATCTACAACTGCTGGTCCA-BHQ1 | [15] |
|  | *cpa* | α toxin | F, AAAAGAAAGATTTGTAAGGCGCTTAT；  R, CCCAAGCGTAGACTTTAGTTGATG  P, FAM-TGCCGCGCTAGCAACTAGCCTATGG-BHQ1 | [16] |
|  | *plc* | α toxin | F, GCATGAGTCATAGTTGGGATGATT；  R, CCTGCTGTTCCTTTTTGAGAGTTAG  P, FAM-TGCAGCAAAGGTAACTT-MGB-NFQ | [17] |
| *Salmonella enterica* | *invA^b^* | invasion protein | F, GTGAAATTATCGCCACGTTCGGGCAA；  R, CCCAAGCGTAGACTTTAGTTGATG  P, FAM-TTATTGGCGATAGCCTGGCGGTGGGTTTTGTTG-BHQ1 | modified  [18] |
| *Salmonella* | *invA-*Sal | invasion protein | F, CGTTTCCTGCGGTACTGTTAATT；  R, TCATCGCACCGTCAAAGGAACC  P, FAM-CCACGCTCTTTCGTCT-MGB-NFQ | [17] |
|  | *invA* | invasion protein | F, TCGGGCAATTCGTTATTGG；  R, GATAAACTGGACCACGGTGACA  P, FAM-AAGACAACAAAACCCACCGC-MGB-NFQ | [10] |
| *Listeria monocytogenes* | *Listeriolysin O* | virulence factor | F, TGCAAGTCCTAAGACGCCA；  R, CACTGCATCTCCGTGGTATACTAA  P, FAM-CGATTTCATCCGCGTGTTTCTTTTCG-BHQ1 | [18] |
|  | *iap* | invasion associated  protein p60 | F, AGTGCTATTATTGCTGAAGCTCAAAA；  R, TCCGTTACCACCCCATGAAT  P, FAM-CACCTTGGAAAAGC-MGB-NFQ | [17] |
|  | *hlyA* | virulence factor | F, ACTTCGGCGCAATCAGTGA；  R, TTGCAACTGCTCTTTAGTAACAGCTT  P, FAM-TGAACCTACAAGACCTTCCAGATTTTTCGGC-BHQ1 | [19] |
| enterohemorrhagic *Escherichia coli* O157:H7 | *stx1* | Shiga toxin 1 | F, GACTGCAAAGACGTATGTAGATTCG；  R, ATCTATCCCTCTGACATCAACTGC  P, FAM-TGAATGTCATTCGCTCTGCAATAGGTACTC-BHQ1 | [18] |
|  | *stx2* | Shiga toxin 2 | F, ATTAACCACACCCCACCG；  R, GTCATGGAAACCGTTGTCAC  P, FAM-CAGTTATTTTGCTGTGGATATACGAGGGCTTG-BHQ1 | [18] |
|  | *eae* | intimin | F, GTAAGTTACACTATAAAAGCACCGTCG；  R, TCTGTGTGGATGGTAATAAATTTTTG  P, FAM-AAATGGACATAGCATCAGCATAATAGGCTTGCT-BHQ1 | [18] |
| Shiga-toxigenic *E. coli* | *stx1* | Shiga toxin 1 | F, ACTTCTCGACTGCAAAGACGTATG；  R, ACAAATTATCCCCTGWGCCACTATC  P, FAM-CTCTGCAATAGGTACTCCA-MGB-NFQ | [14] |
|  | *stx2* | Shiga toxin 2 | F, CCACATCGGTGTCTGTTATTAACC；  R, GGTCAAAACGCGCCTGATAG  P, FAM-TTGCTGTGGATATACGAGG-MGB-NFQ | [17] |
| enteropathogenic *E. coli* | *eae* | intimin | F, CATTGATCAGGATTTTTCTGGTGATA；  R, CTCATGCGGAAATAGCCGTTA  P, FAM-ATACTGGCGAGACTATTTCAA-MGB-NFQ | [20] |
|  | *bfpA* | Bundle-forming pili | F, TGGTGCTTGCGCTTGCT；  R, CGTTGCGCTCATTACTTCTG  P, FAM-CAGTCTGCGTCTGATTCCAA-MGB-NFQ | [10] |
| enteroaggregative *E. coli* | *aaiC* | type VI secretion system | F, ATTGTCCTCAGGCATTTCAC；  R, ACGACACCCCTGATAAACAA  P, FAM-TAGTGCATACTCATCATTTAAG-MGB-NFQ | [14] |
|  | *aatA* | *antiaggregation protein transporter* | F, CTGGCGAAAGACTGTATCAT；  R, TTTTGCTTCATAAGCCGATAGA  P, FAM-TGGTTCTCATCTATTACAGACAGC-MGB-NFQ | [10] |
| enterotoxigenic *E. coli* | *elt* | heat-labile enterotoxin | F, TTCCCACCGGATCACCAA；  R, CAACCTTGTGGTGCATGATGA  P, FAM-CTTGGAGAGAAGAACCCT-MGB-NFQ | [14] |
| *Shigella* spp. | *ipaH* | invasion plasmid antigen H | F, CCTTTTCCGCGTTCCTTGA；  R, CGGAATCCGGAGGTATTGC  P, FAM-CGCCTTTCCGATACCGTCTCTGCA-BHQ1 | [21] |
|  | *ipaH-*Shig1 | invasion plasmid antigen H | F, CTTGACCGCCTTTCCGATA；  R, AGCGAAAGACTGCTGTCGAAG  P, FAM-AACAGGTCGCTGCATGGCTGGAA-BHQ1 | [19] |
| *Giardia lamblia* | β-Giardin P434 (P1) | β-Giardin gene assemblage A | F, CCTCAAGAGCCTGAACGATCTC；  R, AGCTGGTCGTACATCTTCTTCCTT  P, FAM-TTCTCCGTGGCAATGCCCGTCT-BHQ1 | [22] |
|  | β-Giardin P434 (H3) | β-Giardin gene assemblage B | F, CCTCAAGAGCCTGAACGACCTC；  R, AGCTGGTCATACATCTTCTTCCTC  P, FAM-TTCTCCGTGGCGATGCCTGTCT-BHQ1 | [22] |
|  | β-Giardin P241 | β-Giardin | F, CATCCGCGAGGAGGTCAA；  R, GCAGCCATGGTGTCGATCT  P, FAM-AAGTCCGCCGACAACATGTACCTAACGA-BHQ1 | [18] |
|  | 18S rRNA | 18S rRNA | F, GACGGCTCAGGACAACGGTT；  R, TTGCCAGCGGTGTCCG  P, FAM-CCCGCGGCGGTCCCTGCTAG-BHQ1 | [23] |
| *Cryptosporidium parvum* | *COWP* | oocystatin | F, CAAATTGATACCGTTTGTCCTTCTG；  R, GGCATGTCGATTCTAATTCAGCT  P, FAM-TGCCATACATTGTTGTCCTGACAAATTGAAT-BHQ1 | [22] |
| *Cryptosporidium species* human-pathogenic | 18S rRNA | 18S rRNA | F, GGGTTGTATTTATTAGATAAAGAACCA；  R, AGGCCAATACCCTACCGTCT  P, FAM-TGACATATCATTCAAGTTTCTGAC-MGB-NFQ | [17] |
| *Entamoeba histolytica* | 18S rRNA | 18S rRNA | F, ATTGTCGTGGCATCCTAACTCA；  R, GCGGACGGCTCATTATAACA  P, FAM-TCATTGAATGAATTGGCCATTT-MGB-NFQ | [23] |
| *Clostridium difficile* | *tcdB* | toxin B | F, GGTATTACCTAATGCTCCAAATAG；  R, TTTGTGCCATCATTTTCTAAGC  P, FAM-CCTGGTGTCCATCCTGTTTC-MGB-NFQ | [10] |
|  | *cdtA^c^* | cytolethal distending toxin A | F, GATCTGGTCCTCAAGAATTTGGTT；  R, GCTTGTCCTTCCCATTTTGATT  P, FAM-AACTCTTACTTCCCCTGAAT-MGB-NFQ | modified  [24] |
|  | *cdtB* | cytolethal distending toxin B | F, AAAAGCTTCAGGTTCTTTTGACAAG；  R, TGATCAGTAGAGGCATGTTCATTTG  P, FAM-CAAGAGATCCGTTAGTTGCAGCATATCCAATTGT-BHQ1 | [24] |
|  | *tcdA* | toxin A | F, CAGTCGGATTGCAAGTAATTGACAAT；  R, AGTAGTATCTACTACCATTAACAGTCTGC  P, FAM-TTGAGATGATAGCAGTGTCAGGATTG-BHQ1 | [25] |
| *Aeromonas hydrophilia* | *aha1* | adherence factors | F, ACCGCTGCTCATTACTCTGATG；  R, CCAACCCAGACGGGAAGAA  P, FAM-TGATGGTGAGCTGGTTG-MGB-NFQ | [17] |
| *Bacillus cereus/B. thuringiensis* | *bceT ^a^* | enterotoxin | F, AATTACATTACCAGGACGTGCTTACTT；  R, TCCAAGCTGATTGGAATAGTTCATAA  P, FAM-CAAGTTGGGAATAATG-MGB-NFQ | [17] |
| *Cronobacter* spp. | *ITS* | G operon | F, CCGGAACAAGCTGAAAATTGA；  R, TCTTCGTGCTGCGAGTTTG  P, FAM-ACTCTGACACACCGCGCATTCCTG-BHQ1 | [26] |
|  | *MMS* | macromolecular synthesis operon | F, GGGATATTGTCCCCTGAAACAG；  R, CGAGAATAAGCCGCGCATT  P, FAM-AGAGTAGTAGTTGTAGAGGCCGTGCTTCCGAAAG-BHQ1 | [27] |
| *Helicobacter pylori* | *vacA* | vacuolating cytotoxin | F, GAATTCCCTAACAAGGAATACGACTT；  R, CCAATCCCAACCTCCATCAA  P, FAM-ACAGATCCCTTTTATCC-MGB-NFQ | [17] |
|  | *urea* | urease subunit α | F, CGTGGCAAGCATGATCCAT；  R, GGGTATGCACGGTTACGAGTTT  P, FAM-TCAGGAAACATCGCTTCAATACCCACTT-BHQ1 | [28] |
|  | 16S rRNA | 16S rRNA | F, CTCATTGCGAAGGCGACCT；  R, TCTAATCCTGTTTGCTCCCCA  P, FAM-ATTACTGACGCTGATTGCGCGAAAGC-BHQ1 | [29] |
| *Staphylococcus aureus* | *sec* | enterotoxin C | F, CGTATTAGCAGAGAGCCAACCA；  R, GTGAATTTACTCGCTTTGTGCAA  P, FAM-ACCCTACGCCAGATGA-MGB-NFQ | [17] |
|  | *nuc* | thermostable nuclease | F, CGCTACTAGTTGCTTAGTGTTAACTTTAGTTG；  R, TGCACTATATACTGTTGGATCTTCAGAA  P, FAM-TGCATCACAAACAGATAACGGCGTAAATAGAAG-BHQ1 | [30] |
|  | *tufa* | translation elongation factor | F, CATGGTTGACGATGAAGAATTATTAGA；  R, TGGGAAGTCATATTCGCTTAATAAGTC  P, FAM-AGTAGAAATGGAAGTTCG-MGB-NFQ | [31] |
| *Pseudomonas aeruginosa* | *ecfX* | extracytoplasmic function σ factor | F, CGCATGCCTATCAGGCGTT；  R, GAACTGCCCAGGTGCTTGC  P, FAM-ATGGCGAGTTGCTGCGCTTCCT-BHQ1 | [27] |
|  | *gyrB* | DNA gyrase subunit B | F, CCTGACCATCCGTCGCCACAAC；  R, CGCAGCAGGATGCCGACGCC  P, FAM-CCGTGGTGGTAGACCTGTTCCCAGACC-BHQ1 | [27] |
|  | *regA* | regulator for exotoxin A | F, TGCTGGTGGCACAGGACAT；  R, TTGTTGGTGCAGTTCCTCATTG  P, FAM-CAGATGCTTTGCCTCAA-MGB-NFQ | [17] |
| *Legionella pneumophila* | *mip* | macrophage infectivity potentiator | F, AAAGGCATGCAAGACGCTATG；  R, GAAACTTGTTAAGAACGTCTTTCATTTG  P, FAM-TGGCGCTCAATTGGCTTTAACCGA-BHQ1 | [32] |
|  | *mip*-Lpne | macrophage infectivity potentiator | F, ACCGATGCCACATCATTAGCT；  R, CCAAATCGGCACCAATGC  P, FAM-CAGACAAGGATAAGTTGTC-MGB-NFQ | [17] |
| *Leptospira* spp. | *lipL32* | lipoprotein | F, AAGCATTACCGCTTGTGGTG；  R, GAACTCCCATTTCAGCGATT  P, FAM-AAAGCCAGGACAAGCGCCG-BHQ1 | [18] |
| *Mycobacterium tuberculosis* | *IS6110* | insertion sequence 6110 | F, AGACGTTATCCACCATAC；  R, AGTGCATTGTCATAGGAG  P, FAM-TCTCAGTACACATCGATCCGGT-BHQ1 | [33] |
|  | *IS6110*-p | insertion sequence 6110 | F, CCGAGGCAGGCATCCA；  R, GATCGTCTCGGCTAGTGCATT  P, FAM-TCGGAAGCTCCTATGAC-MGB-NFQ | [34] |
| *Mycobacterium avium* subsp. *paratuberculosis* | *IS900* | insertion sequence 900 | F, AATGACGGTTACGGAGGTGGT；  R, GCAGTAATGGTCGGCCTTACC  P, FAM-TCCACGCCCGCCCAGACAGGTTG-BHQ1 | [18] |
| *Acanthamoeba* spp. | 18S rRNA | 18S rRNA | F, CGACCAGCGATTAGGAGACG；  R, CCGACGCCAAGGACGAC  P, FAM-TGAATACAAAACACCACCATCGGCGC-BHQ1 | [27] |
| *Klebsiella pneumoniae* | *phoE* | outer membrane phosphate porin | F, CCTGGATCTGACCCTGCAGTA；  R, CCGTCGCCGTTCTGTTTC  P, FAM-CAGGGTAAAAACGAAGGC-MGB-NFQ | [17] |
| *Streptococcus pneumoniae* | *lytA* | autocytolysin | F, ACGCAATCTAGCAGATGAAGC；  R, TGTTTGGTTGGTTATTCGTGC  P, FAM-TTTGCCGAAAACGCTTGATACAGGG-BHQ1 | [35] |
|  | *lytA*-CDC | autocytolysin | F, ACGCAATCTAGCAGATGAAGCA；  R, TCGTGCGTTTTAATTCCAGCT  P, FAM-GCCGAAAACGCTTGATACAGGGAG-BHQ1 | [36] |
|  | *psaA* | pneumococcal surface adhesion | F, GCCCTAATAAATTGGAGGATCTAATGA；  R, GACCAGAAGTTGTATCTTTTTTTCCG  P, FAM-CTAGCACATGCTACAAGAATGATTGCAGAAAGAAA-BHQ1 | [36] |
|  | *cpsA* | capsular polysaccharide biosynthesis | F, GCTGTTTTAGCAGATAGTGAGATCGA；  R, TCCCAGTCGGTGCTGTCA  P, FAM-AATGTTACGCAACTGACGAG-MGB-NFQ | [37] |
| *Neisseria gonorrhoeae* | *opa* | opacity protein | F, GTTGAAACACCGCCCGG；  R, CGGTTTGACCGGTTAAAAAAAGAT  P, FAM-CCCTTCAACATCAGTGAAA-MGB-NFQ | [38] |

^a^ Marker gene can simultaneously target *Bacillus cereus* and *B. thuringiensis*. Therefore, we revised the target to be *Bacillus cereus*/*B. thuringiensis.*

*^b, c^* Oligonucleotide sequences of the primers were modified on the basis of BLASTn alignments.

**Table S4** Standard curve data for pathogenic marker genes.

| **Marker Gene** | **Amplification Efficiency** | **Standard Curve** | **Determination Coefficient** |
| --- | --- | --- | --- |
| *Acanthamoeba* spp. 18S rRNA | 99.96 | = -3.195x + 40.11 | *R*² = 0.990 |
| *Aeromonas hydrophilia* *aha1* | 103.63 | = -3.300x + 39.35 | *R*² = 0.987 |
| *Bacillus cereus bceT* | 96.33 | = -3.222x + 40.16 | *R*² = 0.995 |
| Bact2 | 109.13 | = -3.121x + 44.10 | *R*² = 0.994 |
| *Campylobacter coli cdtA* | 98.31 | = -3.912x + 44.21 | *R*² = 0.996 |
| *Campylobacter coli ceuE* | 93.69 | = -3.204x + 37.50 | *R*² = 0.989 |
| *Campylobacter jejuni- C. coli cadF* | 91.76 | = -3.567x + 40.67 | *R*² = 0.988 |
| *Campylobacter jejuni hipO* | 99.71 | = -3.406x + 40.01 | *R*² = 0.989 |
| *Campylobacter lari pepT* | 97.71 | = -3.296x + 38.41 | *R*² = 0.989 |
| *Clostridium difficile tcdA* | 93.22 | = -3.578x + 42.56 | *R*² = 0.999 |
| *Clostridium difficile tcdB* | 103.18 | = -3.248x + 36.88 | *R*² = 0.999 |
| *Clostridium perfringens cpa* | 98.72 | = -3.353x + 40.54 | *R*² = 0.999 |
| *Clostridium perfringens plc* | 108.78 | = -3.172x + 39.49 | *R*² = 0.993 |
| *Cronobacter* spp. ITS | 93.63 | = -3.572x + 39.37 | *R*² = 0.992 |
| *Cronobacter* spp. MMS | 95.15 | = -3.073x + 39.90 | *R*² = 0.991 |
| *Cryptosporidium* human-pathogenic species 18S rRNA | 104.03 | = -3.023x + 37.45 | *R*² = 0.991 |
| *Cryptosporidium parvum* COWP | 92.28 | = -3.536x + 41.73 | *R*² = 0.992 |
| *Entamoeba histolytica* 18S rRNA | 92.71 | = -3.414x + 40.69 | *R*² = 0.990 |
| enteroaggregative *E. coli aaiC* | 90.69 | = -3.236x + 37.30 | *R*² = 0.998 |
| enteropathogenic *E.coli bfpA* | 91.15 | = -3.468x + 38.59 | *R*² = 0.991 |
| enteropathogenic *E.coli eae* | 100.33 | = -3.669x + 39.76 | *R*² = 0.968 |
| enterotoxigenic *E. coli elt* | 93.95 | = -3.461x + 39.06 | *R*² = 0.993 |
| *Escherichia coli* O157:H7 *stx1* | 99.49 | = -3.334x + 40.27 | *R*²= 0.998 |
| *Giardia lamblia* β-Giardin P241 | 96.76 | = -3.663x + 41.28 | *R*² = 0.978 |
| *Giardia lamblia* β-Giardin P434 (H3) | 100.67 | = -3.345x + 40.57 | *R*² = 0.993 |
| *Giardia lamblia* β-Giardin P434 (P1) | 99.42 | = -3.321x + 39.09 | *R*² = 0.983 |
| *Giardia lamblia* 18S rRNA | 97.24 | = -3.10x + 38.00 | *R*² = 0.982 |
| *Helicobacter pylori* 16S rRNA | 97.47 | = -3.279x + 38.99 | *R*² = 0.993 |
| *Helicobacter pylori ureA* | 101.05 | = -3.297x + 39.83 | *R*² = 0.997 |
| *Helicobacter pylori vacA* | 94.62 | = -3.458x + 41.43 | *R*² = 0.998 |
| *Klebsiella pneumoniae phoE* | 102.17 | = -3.154x + 40.38 | *R*² = 0.992 |
| *Legionella pneumophila mip* | 99.05 | = -3.403x + 43.27 | *R*² = 0.993 |
| *Legionella pneumophila mip Lpne* | 94.99 | = -3.474x + 43.37 | *R*² = 0.988 |
| *Legionella* spp. 23S rRNA | 103.00 | = -3.284x + 39.83 | *R*² = 0.981 |
| *Leptospira* spp. *lipL32* | 100.37 | = -3.416x + 38.58 | *R*² = 0.989 |
| *Listeria monocytogenes hlyA* | 99.63 | = -3.331x + 38.33 | *R*² = 0.995 |
| *Listeria monocytogenes iap* | 104.12 | = -3.227x + 40.09 | *R*² = 0.999 |
| *Listeria monocytogenes* Listeriolysin O | 108.24 | = -3.516x + 41.42 | *R*² = 0.994 |
| *Mycobacterium avium* subsp. paratuberculosis IS900 | 99.29 | = -3.577x + 41.71 | *R*² = 0.990 |
| *Mycobacterium tuberculosis* IS6110-p | 98.88 | = -3.254x + 37.72 | *R*² = 0.993 |
| *Neisseria gonorrhoeae opa* | 93.62 | = -3.485x + 41.65 | *R*² = 0.999 |
| *Pseudomonas aeruginosa ecfX* | 91.15 | = -3.669x + 43.87 | *R*² = 0.971 |
| *Pseudomonas aeruginosa gyrB* | 97.35 | = -3.368x + 40.31 | *R*² = 0.973 |
| *Pseudomonas aeruginosa regA* | 94.92 | = -3.409x + 41.16 | *R*² = 0.994 |
| *Salmonella enterica invA* | 98.13 | = -3.345x + 41.23 | *R*² = 0.966 |
| *Salmonella invA* | 104.12 | = -3.237x + 38.82 | *R*² = 0.982 |
| *Salmonella invA Sal* | 103.22 | = -3.363x + 40.77 | *R*² = 0.994 |
| Shiga-toxigenic *E. coli stx1* | 99.05 | = -3.349x + 41.06 | *R*²² = 0.996 |
| Shiga-toxigenic *E. coli stx2* | 98.84 | = -3.164x + 39.48 | *R*² = 0.978 |
| *Shigella ipaH* | 105.58 | = -3.195x + 40.09 | *R*² = 0.997 |
| *Shigella ipaH Shig1* | 97.16 | = -3.392x + 40.35 | *R*² = 0.997 |
| *Staphylococcus aureus nuc* | 102.26 | = -3.269x + 39.48 | *R*² = 0.994 |
| *Staphylococcus aureus sec* | 101.56 | = -3.645x + 42.49 | *R*² = 0.968 |
| *Staphylococcus aureus tufA* | 99.83 | = -3.311x + 39.74 | *R*² = 0.995 |
| *Streptococcus pneumoniae cpsA* | 93.95 | = -3.476x + 40.83 | *R*² = 0.997 |
| *Streptococcus pneumoniae lytA* | 96.45 | = -3.114x + 38.33 | *R*² = 0.979 |
| *Vibrio cholerae ctxA* | 101.62 | = -3.284x + 38.59 | *R*² = 0.995 |
| *Vibrio cholerae- V. parahaemolyticus toxR* | 114.35 | = -3.306x + 37.81 | *R*² = 0.991 |
| *Vibrio cholerae-total ompW* | 107.23 | = -3.470x + 41.24 | *R*² = 0.990 |
| *Vibrio cholerae-toxigenic ctxA* | 100.04 | = -3.295x + 38.71 | *R*² = 0.977 |

**Reference**

1. Paes J, Kepler R, Gonalves RF, Berte FK, Virginio VG, Benitez LB, et al. Amoebal coculture and enrichment methods as a proposal for water quality control in Brazil. Acta Trop. 2021;223:106074.

2. Wittmann J, Dreiseikelmann B, Rohde C, Rohde M, Sikorski J. Isolation and characterization of numerous novel phages targeting diverse strains of the ubiquitous and opportunistic pathogen Achromobacter xylosoxidans. PLoS One. 2014;9(1):e86935.

3. Al-Asadi SA, Al-Kahachi RES, Alwattar WMA, Bootwala J, MA. S. Genomic insights into *Achromobacter mucicolens* IA antibiotic resistance. Microbiol Spectr. 2022;10(2):e0191621.

4. Li G, Yang L, Zhang T, Guo X, Qin J, Cao Y, et al. Complete genome sequence of *Achromobacter spanius* type strain *DSM 23806T*, a pathogen isolated from human blood. J Glob Antimicrob Resist. 2018;14:1-3.

5. Buss SN, Starlin R, Iwen PC. Bacteremia caused by *Microbacterium binotii* in a patient with sickle cell anemia. J Clin Microbial. 2014;52(1):379-81.

6. Sridhar S, Wang AYM, Chan JFW, Yip CCY, Lau SKP, Woo PCY, et al. First report of human infection by *Agromyces mediolanus*, a gram-positive organism found in soil. J Clin Microbial. 2015;53(10):3377-9.

7. Aguilera-Arreola MG, Ostria-Hernández ML, Albarrán-Fernández E, Juárez-Enriquez SR, Majalca-Martínez C, Rico-Verdín B, et al. Correct identification of *Ochrobactrum anthropi* from blood culture using 16r RNA sequencing: A first case report in an immunocompromised patient in Mexico. Front Med. 2018;5:205.

8. Looney WJ, Narita M, Mühlemann K. *Stenotrophomonas maltophilia*: an emerging opportunist human pathogen. Lancet Infect Dis. 2009;9(5):312-23.

9. Timothy L, Richard W, Egon O, Alan H. First report of *Pseudomonas nitroreducens* cultured from the lungs of a patient with pneumonia. BMJ Case Rep. 2021;14(5):e241327.

10. Liu J, Gratz J, Amour C, Kibiki G, Becker S, Janaki L, et al. A laboratory-developed TaqMan array card for simultaneous detection of 19 enteropathogens. J Clin Microbial. 2013;51(2):472-80.

11. Best EL, Powell EJ, Craig S, Grant KA, Frost JA. Applicability of a rapid duplex real-time PCR assay for speciation of *Campylobacter jejuni* and *Campylobacter coli* directly from culture plates. FEMS Microbiol Lett. 2010;229(2):237-41.

12. He Y, Yao X, Gunther NW, Xie Y, Tu SI, Shi X. Simultaneous detection and differentiation of *Campylobacter jejuni*, *C. coli*, and *C. lari* in chickens using a multiplex real-time PCR assay. Food anal methods. 2010;3(4):321-9.

13. Blackstone GM, Nordstrom JL, Bowen MD, Meyer RF, Imbro P, Paola D. Use of a real time PCR assay for detection of the ctx A gene of *Vibrio cholerae* in an environmental survey of Mobile Bay. J Microbiol Methods. 2007;68(2):254-9.

14. Bliem R, Schauer S, Plicka H, Obwaller A, Sommer R, Steinrigl A, et al. A novel triplex quantitative PCR strategy for quantification of toxigenic and nontoxigenic *Vibrio cholerae* in aquatic environments. Appl Environ Microbiol. 2015;81(9):3077-85.

15. Minamoto Y, Dhanani N, Markel ME, Steiner JM, Suchodolski JS. Prevalence of *Clostridium perfringens*, *Clostridium perfringens* enterotoxin and dysbiosis in fecal samples of dogs with diarrhea. Veterin Microbiol. 2014;174(3-4):463-73.

16. Mcauley CM, Mcmillan K, Moore SC, Fegan N, Fox EM. Prevalence and characterization of foodborne pathogens from Australian dairy farm environments. J Dairy Sci. 2014;97(12):7402-12.

17. Lee DY, Shannon K, Beaudette LA. Detection of bacterial pathogens in municipal wastewater using an oligonucleotide microarray and real-time quantitative PCR. J Microbiol Methods. 2006;65(3):453-67.

18. Hein I, Flekna G, Krassnig M, Wagner M. Real-time PCR for the detection of *Salmonella spp.* in food: An alternative approach to a conventional PCR system suggested by the FOOD-PCR project. J Microbiol Methods. 2006;66(3):538-47.

19. Garrido A, Chapela MJ, Román B, Ferreira M, Lago J, Vieites JM, et al. Development of a multiplex real-time PCR method for simultaneous detection of *Salmonella enterica*, *Shigella flexneri* and *Listeria monocytogenes* in processed food samples. Europ Food Res Technol. 2012;234(4):571-80.

20. Bugarel M, Beutin L, Fach P. Low-density macroarray targeting non-locus of enterocyte effacement effectors (nle genes) and major virulence factors of *Shiga toxin-producing Escherichia coli* (STEC): a new approach for molecular risk assessment of STEC isolates. Appl Environ Microbiol. 2010;76(1):203-11.

21. Thiem VD, Sethabutr O, Seidlein LV, Tung TV, Canh DG, Chien BT, et al. Detection of *Shigella* by a PCR assay targeting the ipaH gene suggests increased prevalence of *Shigellosis* in Nha Trang, Vietnam. J Clin Microbial. 2004;42(5):2031-5.

22. Guy RA, Payment P, Krull UJ, Horgen PA. Real-time PCR for quantification of *Giardia* and *Cryptosporidium* in environmental water samples and sewage. Appl Environ Microbiol. 2003;69(9):5178-85.

23. Verweij JJ, Blange RA, Templeton K, Schinkel J, Brienen E, Rooyen MV, et al. Simultaneous detection of *Entamoeba histolytica*, *Giardia lamblia*, and *Cryptosporidium parvum* in fecal samples by using multiplex real-time PCR. J Clin Microbial. 2004;42(3):1220-3.

24. Wroblewski D, Hannett GE, Bopp DJ, Dumyati GK, Halse TA, Dumas NB, et al. Rapid molecular characterization of *Clostridium difficile* and assessment of populations of *C. difficile* in stool specimens. J Clin Microbial. 2009;47(7):2142-8.

25. Hiroyuki K, Takafumi S, Agata G, Hiroshi M, Takuya A. Development of TaqMan-based quantitative PCR for sensitive and selective detection of toxigenic *Clostridium difficile* in human stools. PLoS One. 2014;9(10):e111684.

26. Yin L, Cai X, Xia Z, Gao Q, Huang X. Real time PCR using TaqMan and SYBR Green for detection of *Enterobacter sakazakii* in infant formula. J Microbiol Methods. 2006;65(1):21-31.

27. Xiang W, Zhu C, Xu X, Zhou G. Real-time PCR with internal amplification control for the detection of *Cronobacter* spp. *(Enterobacter sakazakii)* in food samples. Food Control. 2012;25(1):144-9.

28. Schabereiter-Gurtner C, Hirschl AM, Dragosics B, Hufnagl P, Puz S, Kovach Z, et al. Novel real-time PCR assay for detection of *Helicobacter pylori* infection and simultaneous clarithromycin susceptibility testing of stool and biopsy specimens. J Clin Microbial. 2004;42(10):4512-8.

29. Minami J, Ishige I, Eishi Y, Yamada T, Koike M, Ohkusa T, et al. Gastric mucosal density of Helicobacter pylori estimated by real-time PCR compared with results of urea breath test and histological grading. J Med Microbial. 2002;51(4):305-11.

30. Alarcón B, Vicedo B, R. A. PCR-based procedures for detection and quantification of *Staphylococcus aureus* and their application in food. J Appl Microbial. 2010;100(2):352-64.

31. Boardman AK, Campbell J, Wirz H, Sharon A, Sauer-Budge AF. Rapid microbial sample preparation from blood using a novel concentration device. Plos One. 2014;10(2):e0116837.

32. Nazarian EJ, Bopp DJ, Saylors A, Limberger RJ, Musser KA. Design and implementation of a protocol for the detection of *Legionella* in clinical and environmental samples. Diagn Microbiol Infect Dis. 2008;62(2):125-32.

33. Francesca B, Carlton A, Evans L, Rigouts J, Arévalo K. Standardization of a TaqMan-based real-time PCR for the detection of *Mycobacterium tuberculosis-Complex* in human sputum. Am J Trop Med Hyg. 2014;91(4):709-14.

34. Lemaître N, Armand S, Vachée A, Capilliez O, Dumoulin C, RJ. C. Comparison of the real-time PCR Method and the Gen-Probe amplified *Mycobacterium tuberculosis* direct test for detection of *Mycobacterium tuberculosis* in pulmonary and nonpulmonary specimens. J Clin Microbiol. 2004;42(9):4307-9.

35. Mcavin JC, Reilly PA, Roudabush RM, Barnes WJ, Salmen A, Jackson GW, et al. Sensitive and specific method for rapid identification of *Streptococcus pneumoniae* using real-time fluorescence PCR. J Clin Microbial. 2001;39(10):3446-51.

36. Carvalho MDGS, Tondella ML, Mccaustland K, Weidlich L, Mcgee L, Mayer LW, et al. Evaluation and improvement of real-time PCR assays targeting *lytA*, *ply*, and *psaA* genes for detection of *pneumococcal* DNA. J Clin Microbial. 2007;45(8):2460-6.

37. Kuk PH, Jung LH, Wonyong K. Real-time PCR assays for the detection and quantification of *Streptococcus pneumoniae*. FEMS Microbiol Lett. 2010;310(1):48-53.

38. Geraats-Peters C, Brouwers M, Schneeberger PM, Van D, Bruisten SM, Weers-Pothoff G, et al. Specific and sensitive detection of *Neisseria gonorrhoeae* in clinical specimens by real-time PCR. J Clin Microbial. 2005;43(11):5653-9.
